# CAR-T cell therapy in brain malignancies: obstacles in the face of cellular trafficking and persistence

**DOI:** 10.3389/fimmu.2025.1596499

**Published:** 2025-06-19

**Authors:** Serge Yaacoub, Elton Vannoy, Stefanyda Maslova, Abigail Haffey, Khatereh Khorsandi, Natasha Sheybani, Dalia Haydar

**Affiliations:** ^1^ Center for Cancer and Immunology Research, Children’s National Hospital, Washington, DC, United States; ^2^ Department of Biomedical Engineering, University of Virginia, Charlottesville, VA, United States; ^3^ Focused Ultrasound Cancer Immunotherapy Center, University of Virginia, Charlottesville, VA, United States

**Keywords:** CAR-T cells, pediatric brain tumors, trafficking, persistence, cellular exhaustion, tumor microenvironment, focused ultrasound, targeted therapy

## Abstract

Chimeric Antigen Receptor T (CAR-T) cell therapy offers substantial promise for the treatment of brain malignancies, yet its clinical translation remains limited. Tumors such as Glioblastoma Multiforme (GBM), Diffuse Intrinsic Pontine Glioma (DIPG), and Medulloblastoma (MB) are associated with poor prognoses and exhibit limited responsiveness to conventional treatment modalities, including radiotherapy, chemotherapy, and surgical resection. The application of CAR-T cell therapy in these contexts faces significant challenges, primarily in terms of efficient cellular trafficking into the tumor microenvironment and access to heterogeneous tumor regions. Furthermore, CAR-T cell persistence, defined by the long-term survival and functionality of infused cells, remains a critical hurdle in achieving durable therapeutic responses and preventing tumor relapses. This review aims to address the two predominant barriers, trafficking and persistence, by discussing the underlying mechanisms that limit CAR-T cell efficacy in brain tumors, reviewing current strategies aimed at overcoming these challenges, and evaluating novel approaches to enhance the effectiveness of CAR-T therapies in this setting.

## 1 Introduction

Chimeric Antigen Receptor (CAR) T-cell therapies have revolutionized cancer treatment, particularly for hematological malignancies, thus significantly improving patient outcomes compared to traditional chemotherapeutic approaches (1). In recent years, adoptive immunotherapy has demonstrated remarkable promise, culminating in FDA approval for several anti-CD19 CAR therapies, including Kymriah for refractory or relapsed follicular lymphoma, Yescarta for large B-cell lymphoma, Tecartus for mantle cell lymphoma, Breyanzi for large B-cell lymphoma, and Carvykti and Abecma for multiple myeloma (2). These achievements underscore the transformative potential of CAR-T cell therapies and have since catalyzed efforts to apply these innovative approaches to solid tumors, particularly those affecting the brain (3).

However, central nervous system (CNS) tumors present distinct challenges that limit the effectiveness of CAR-T cell therapies (4). The solid tumor microenvironment (TME) in the brain is complex, characterized by immune-suppressive tumor cells, dense extracellular matrices, antigen heterogeneity, and limited trafficking of immune cells (5, 6). These barriers have significantly hindered the progress of CAR T cell therapies for brain tumors, despite promising preclinical findings and numerous clinical trials conducted in both adult and pediatric populations (7). While these trials (**Table 1**) have demonstrated safety and feasibility, particularly with intra-tumoral delivery, therapeutic responses have been less robust compared to those observed in hematological malignancies.

**Table 1:** List of clinical trials pertaining to CAR-T therapies for brain tumors in the adult and pediatric populations (A- Pediatric Population; B- Adult Population).

Population	Clinical trial	Phase	Brain tumor classification	Therapy/Intervention	Delivery method	Results and toxicities
A. Pediatric	NCT06221553	Phase I	DIPG	B7H3 with IL-7Ra signaling CAR T cells	ICV administration with an indwelling CNS catheter	–
	NCT04185038	Phase I	DIPG/Diffuse Midline Glioma and Recurrent/refractory pediatric CNS tumors	B7H3 CAR T cells with EGFRt	ICV administration or intra-tumoral administration with an indwelling CNS catheter	Early indications of improved survival. Evidence of immune activation. AEs included headache, nausea/vomiting, and fever.
	NCT05768880	Phase I	DIPG, DMG, recurrent/refractory pediatric CNS tumors	B7H3 EGFR806 HER2 IL13-Zetakine (Quad) CAR T cells	ICV	–
	NCT03638167	Phase I	EGFR^+^ Recurrent/Refractory Pediatric CNS tumors	EGFR806 CAR T cells with EGFRt	ICV administration or intra-tumoral administration with an indwelling CNS catheter	–
	NCT05298995	Phase I	Pediatric Brain/CNS tumors	Ic9-GD2 CAR T cells	IV	–
	NCT04196413	Phase I	H3K27M^+^ DIPG or spinal DMG	GD2 CAR T cells	IV or ICV	3 of 4 patients showed radiographic and clinical benefit after IV administration. Improved neurological symptoms were also observed. AEs included CRS, ICANS, and TIAN, hydrocephalus, and worsening neurological symptoms.
	NCT04099797	Phase I	DMG, HGG, DIPG, Medulloblastoma	GD2 CAR TO cells or C7R-GD2 CAR T cells	ICV	GD2 CAR T cell cohort showed brief neurological improvement, no toxicity. CCR7-GD2 CAR T cell cohort showed temporary neurological improvement, 2 of 7 DMG patients had a partial response. AEs included CRS and TIAN.
	NCT04510051	Phase I	IL13Ra2^+^ Recurrent/refractory Pediatric Brain tumors	IL13 CAR T cells with CD19t	ICV	–
	NCT03500991	Phase I	HER-2^+^ Recurrent/Refractory Pediatric CNS tumors	HER-2 CAR T cells with EGFRt	Intratumoral administration with an indwelling CNS catheter	Evidence of CNS immune activation in 3 patients. No dose-limiting toxicities.
B. Adult	NCT05366179	Phase I	Glioblastoma	B7H3 CAR T cells	ICV	–
	NCT04077866	Phase I/II	Glioblastoma	B7H3 CAR T cells and Temozolomide	ICV or intra-tumoral with an Ommaya catheter	–
	NCT05474378	Phase I	Recurrent Glioblastoma Multiforme	B7H3 CAR T cells	ICV and/or intra-tumoral administration	–
	NCT05241392	Phase I	Recurrent Glioblastoma	B7H3 CAR T cells	ICV or intra-tumoral with an Ommaya device	One partial and one complete response. AEs included cytokine release syndrome, ICP, headache, epilepsy, vomiting, and pyrexia.
	NCT05752877	N/A	Advanced Glioma	B7H3 or IL-13Rα2 UCAR T cells	Intracranial or intravertebral	–
	NCT05627323	Phase I	MMP2^+^ recurrent or progressive Glioblastoma	CLTX CAR T cells	Intra-tumoral and ICV administration with Rickham catheters	–
	NCT01454596	Phase I/II	EGFRvIII^+^ Glioblastoma or Gliosarcoma	EGFRvIII CAR T cells	IV	No definitive responses. 2 cases of hypoxia leading to 1 death.
	NCT03696030	Phase I	Recurrent Brain Metastasis	HER-2 CAR T cells	ICV	–
	NCT02442297	Phase I	HER2^+^ CNS tumors	HER2 CAR T cells	ICV	–
	NCT01109095	Phase I	Glioblastoma Multiforme	CMV-specific HER2 CAR T cells	IV	1 partial response, 7 short-term stable disease, 3 long-term stable disease.
	NCT04661384	Phase I	Leptomeningeal Glioblastoma, Ependymoma, or Medulloblastoma	IL13Ralpha2 CAR T cells	ICV	–
	NCT02208362	Phase I	Recurrent or Refractory Malignant Glioma	IL13Ralpha2 CAR T cells	Intra-tumoral, intracavitary, or ICV via catheter	Stable disease or better for 50% of patients. 2 partial responses, 1 complete response. AEs included encephalopathy, hypertension, and ataxia.
	NCT05353530	Phase I	CD70^+^ Adult Glioblastoma	8R-70 CAR T cells (modified IL-8R, CD70 targeting)	IV	–

ICV, intracerebroventricular; IV, intravenous; DIPG, diffuse intrinsic pontine glioma; DMG, diffuse midline glioma; CNS, central nervous system; HGG, high grade glioma; AE, adverse event; ICP, increased intracranial pressure; CRS, cytokine release syndrome; ICANS, immune effector cell-associated neurotoxicity; TIAN, tumor inflammation-associated neurotoxicity.

This review will focus on two critical hurdles: (1) the inefficiency of CAR T cell trafficking to the tumor site and the complexities of the CNS TME that impede immune cell infiltration, and (2) the lack of CAR T cell persistence and long-term functionality required for sustained tumor control. We will explore the underlying mechanisms that drive these challenges, evaluate current strategies aimed at overcoming them, and discuss emerging approaches to enhance the therapeutic potential of CAR-T cell therapies for brain malignancies. To our knowledge, this is the first comprehensive review to systematically map the full spectrum of barriers undermining CAR T cell efficacy in solid CNS tumors. Unlike previous reviews, our work uniquely integrates both preclinical and clinical data accumulated over several years, with a specific focus on enhancing CAR T cell persistence and delivery in the brain. While we discuss well-established challenges (including heterogeneous antigen expression, the immunosuppressive tumor microenvironment, anatomical constraints, and safety concerns), we also highlight novel technologies and delivery strategies that have not yet been widely incorporated into brain tumor cell therapy. Additionally, we offer our own perspective on how these emerging tools could be leveraged to overcome current limitations. By synthesizing recent clinical trial data alongside cutting-edge preclinical approaches, we propose targeted, stage-specific solutions to improve the endurance and efficacy of CAR T cells in treating CNS tumors.

## 2 CAR-T cells versus other adoptive T-cell therapies

**Figure 1:**
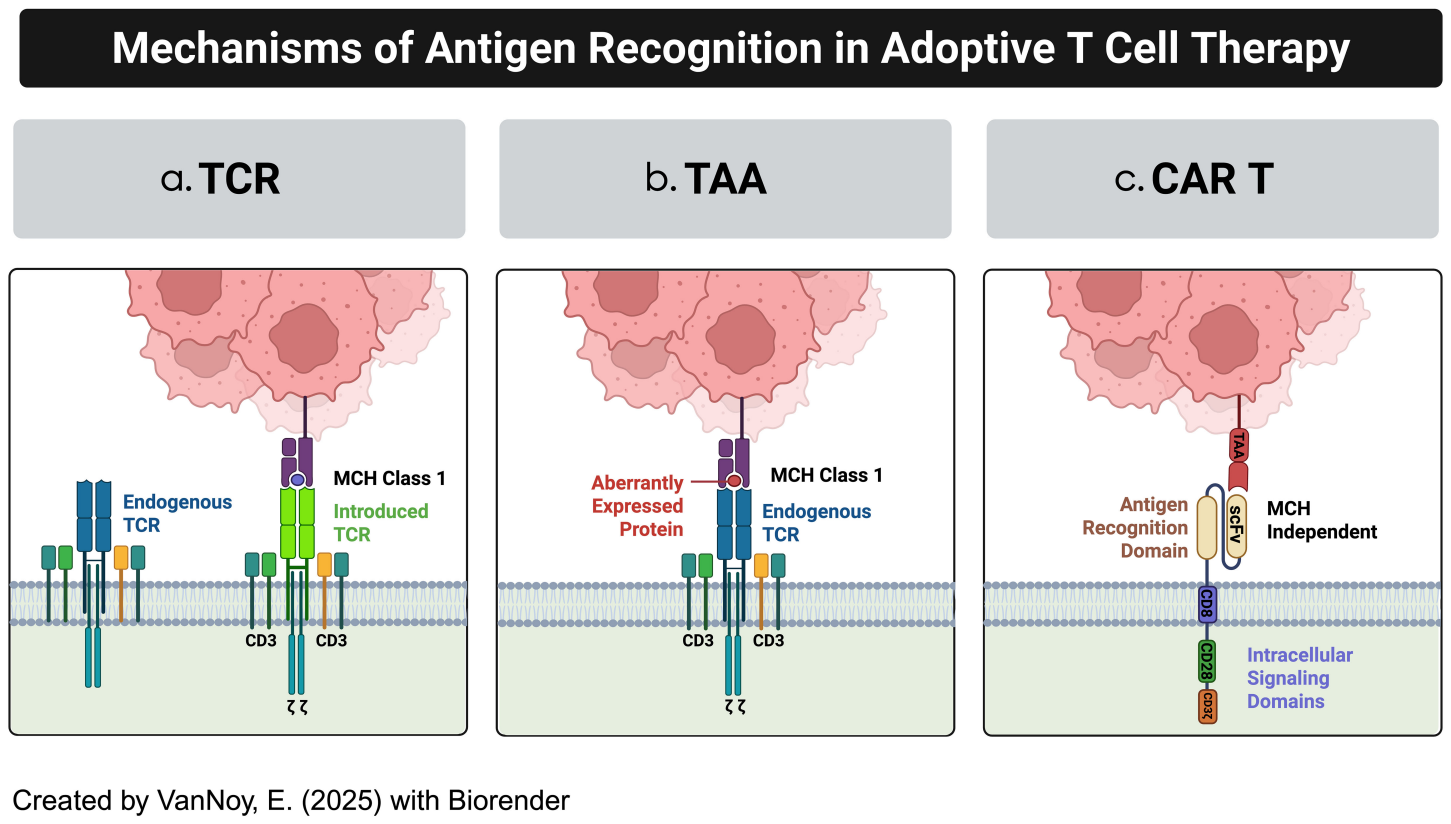
Examples of adoptive T-cell technologies. **(a)** Modified TCR cytotoxic T-cells (CTLs) **(b)** Tumor-associated antigen-specific T cells (TAA-T cells) **(c)** Chimeric antigen receptor modified T cells.

**Figure 2:**
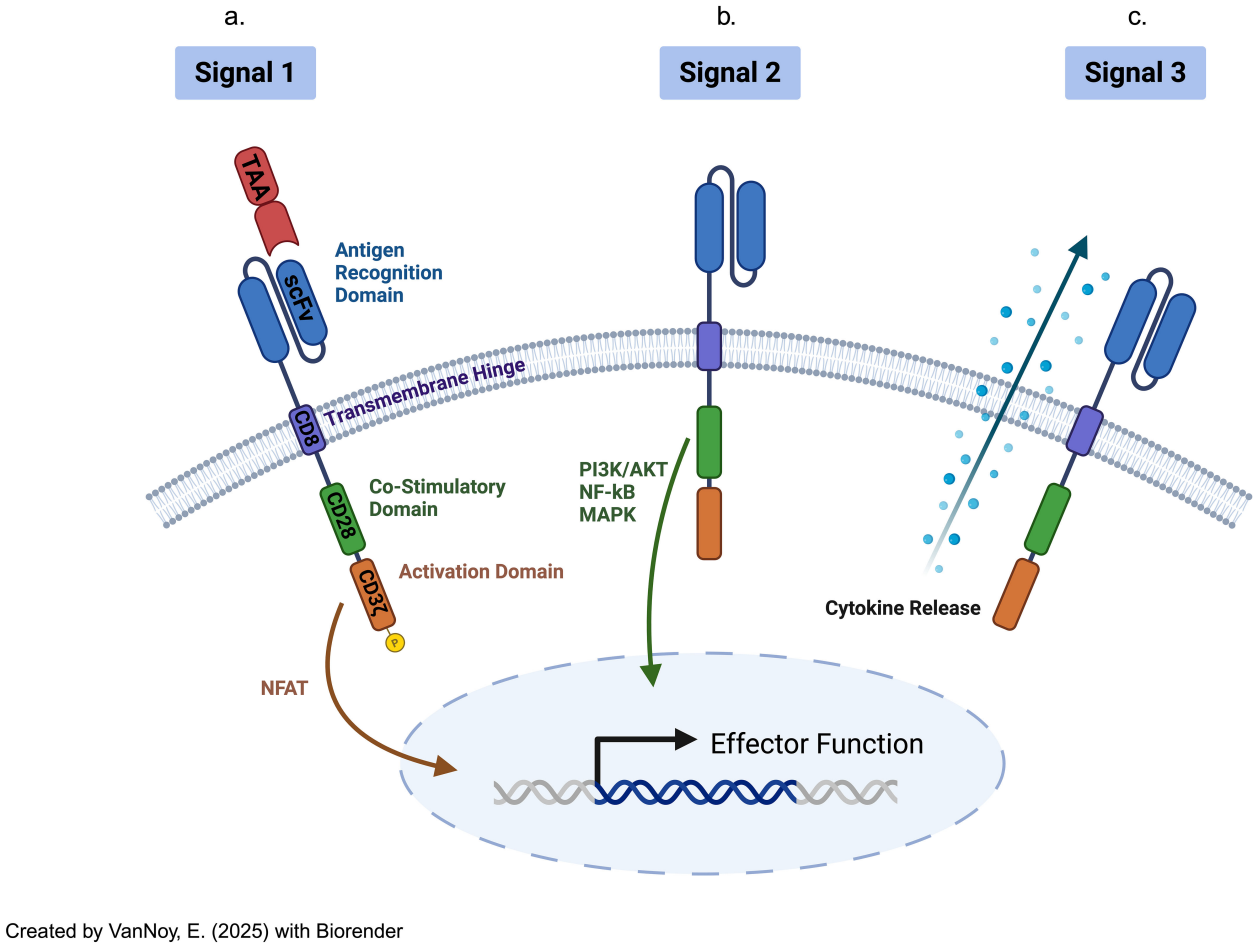
Chimeric antigen receptor-modified T cells have distinct CAR receptors with their own transduction pathways, separate from the TCR-mediated pathways, virtue of their variant extracellular, transmembrane, costimulatory, and intracellular activation domains. The signaling pathway is initiated upon CAR-T cells **(a)** binding to the extracellular ligands (signal 1: "activation"). Then, **(b)** co-stimulation (through signal 2) functions as a necessary co-activation signal, prior to **(c)** cytokine release (signal 3) that supports eventual CAR-T cell differentiation and ultimate activation .

Adoptive T-cell therapies include various approaches such as T-cell receptor (TCR)-engineered T cells, tumor-associated antigen (TAA)-specific T cells, and CAR-T cells, each utilizing distinct mechanisms of action (**Figure 1**) (8, 9):

**a. TCR-Engineered T Cells:** These therapies harness the T-cell receptor’s natural ability to recognize tumor antigens presented on the cell surface by the Major Histocompatibility Complex (MHC) (10). This MHC-dependent mechanism enables targeting intracellular antigens that are processed and displayed (11). However, the need for intact antigen presentation machinery and specific human leukocyte antigen (HLA) haplotypes limits their efficacy, particularly in solid tumors like brain cancers, where antigen presentation is often impaired (12).
**b. TAA-Specific T Cells:** Derived from tumor-infiltrating lymphocytes (TILs) or peripheral T cells, TAA-specific T cells target tumor-associated antigens that are naturally recognized by the immune system (13, 14). While these cells can target a broad range of antigens, their effectiveness is reduced by the immunosuppressive tumor microenvironment and the low frequency of tumor-reactive T cells (15, 16).
**c. CAR T Cells:** CAR T cell therapy represents a significant advancement in adoptive immunotherapy, where T cells are genetically engineered to recognize and kill tumor cells (17). This is achieved by introducing synthetic receptors that consist of an extracellular antigen-binding domain (often from antibodies), a transmembrane domain, and an intracellular signaling domain (17). CAR-T cells uniquely recognize antigens in an MHC-independent manner, allowing them to target surface antigens directly without the need for MHC-mediated antigen presentation (9). Additionally, CAR T cells can be engineered with co-stimulatory domains, cytokine secretion modules, and synthetic circuits to enhance their efficacy, persistence, and resistance to immune suppression (18) (**Figure 2**).

Hence, CAR-T cell therapy stands apart from TCR-engineered and TAA-specific T-cell therapy by offering MHC-independent antigen recognition, enabling direct targeting of extracellular antigens (8, 11, 19). While TCR-based therapies are limited to intracellular antigens and require MHC presentation, CAR-T cells can target a broader range of surface antigens. Furthermore, the extensive engineering capabilities of CAR-T cells, such as incorporating co-stimulatory domains and cytokine modules, provide significant advantages for overcoming challenges like antigen heterogeneity and immune evasion in solid tumors (1, 20).

## 3 Challenges for effective CAR T cell therapy in CNS tumors

**Figure 3:**
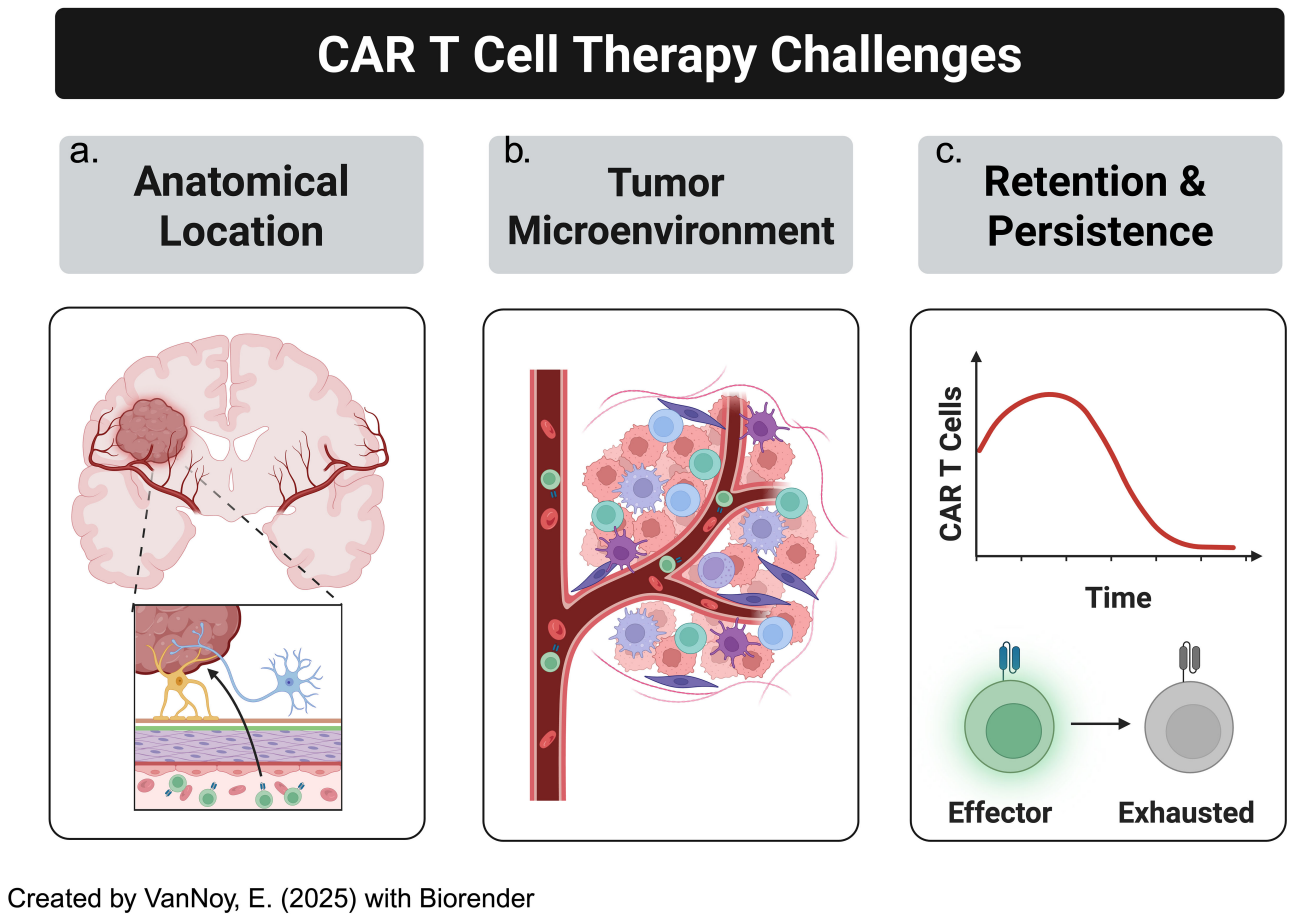
CAR-T cell therapy in solid tumors, particularly brain tumors, has several challenges to overcome. This includes **(a)** location: solid brain tumors are much less accessible than other malignancies, especially hematological malignancies, **(b)** "cold" or "immunosuppressive" tumor microenvironments, **(c)** blunted CAR-T cell persistence.

This section examines the key challenges limiting the success of CAR T cell therapies for CNS tumors, focusing on the anatomical barriers posed by tumor location within the brain, the immunosuppressive TME, and the lack of persistence of CAR T cells in the CNS, all of which hinder effective therapeutic outcomes (**Figure 3**). For clarification, we define pertinent and repetitive terms as the following:

*CNS (Central Nervous System) Tumors*: Neoplastic lesions originating in the brain or spinal cord—including primary and metastatic forms—which face delivery and immunological challenges.

*TME/TIME (Tumor/tumor immune microenvironment):* The local cellular (e.g., myeloid cell populations) and non-cellular (e.g., cytokines, growth factors) milieu within the tumor periphery. It is a major player in dictating immune response efficacy to endogenous and exogenous agents.

*BBB (Blood-brain barrier):* A highly selective barrier that shields the brain from pathogens, toxins, and certain therapeutics, through a dynamic vascular and endothelial framework.

*BTB (Blood-tumor barrier):* The vascular interface surrounding central nervous system tumors that exhibits heterogeneous permeability to drugs and immune-cell infiltration, contrary to the traditional blood-brain barrier (BBB).

### 3.1 Anatomical tumor location within the CNS

The anatomical location of tumors within the CNS significantly impacts the effectiveness of CAR T cell therapy, primarily due to interactions with the blood-brain barrier (BBB), which regulates immune cell access to tumor sites (21, 22) (**Table 2**). The BBB is a selective barrier that restricts the passage of molecules between the bloodstream and the brain, protecting the brain from harmful agents. After the development of the tumor mass with its own vascular supply secondary to cancerous angiogenesis, addressing the “blood-tumor barrier” (BTB) becomes an additional challenge (23). Despite the unregulated nature of the BTB, with its relatively more permissive nature, the underlying BBB intermingled with the BTB forms an additional safety net preventing the free-flowing passage of different cellular and non-cellular agents (24).

**Table 2:** Barriers to CAR T cell trafficking and persistence across pediatric brain tumors.

Variables	GBM	DIPG	MB
Tumor Type	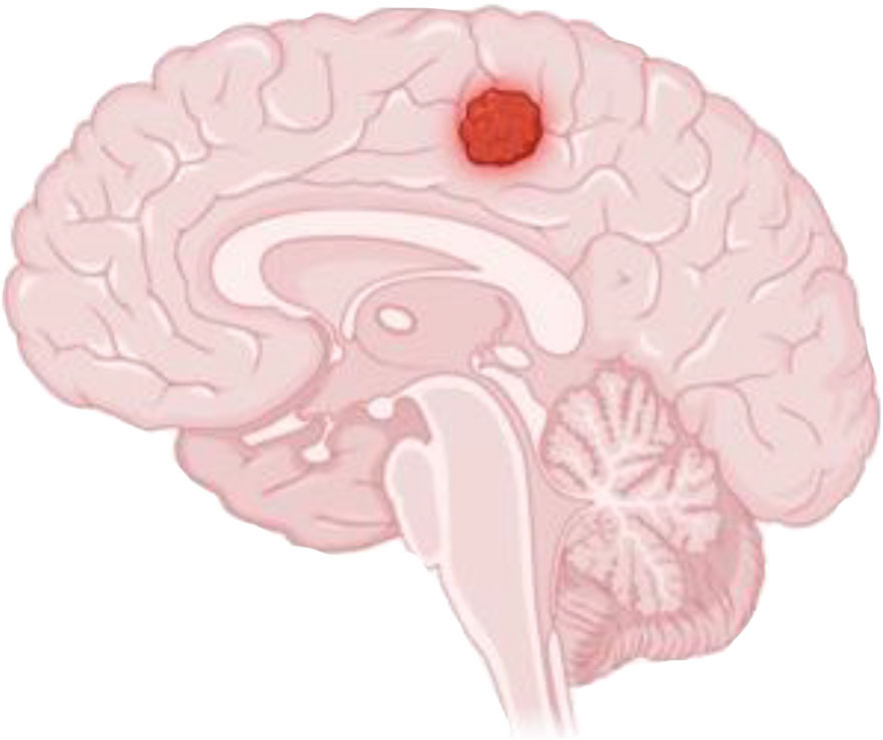	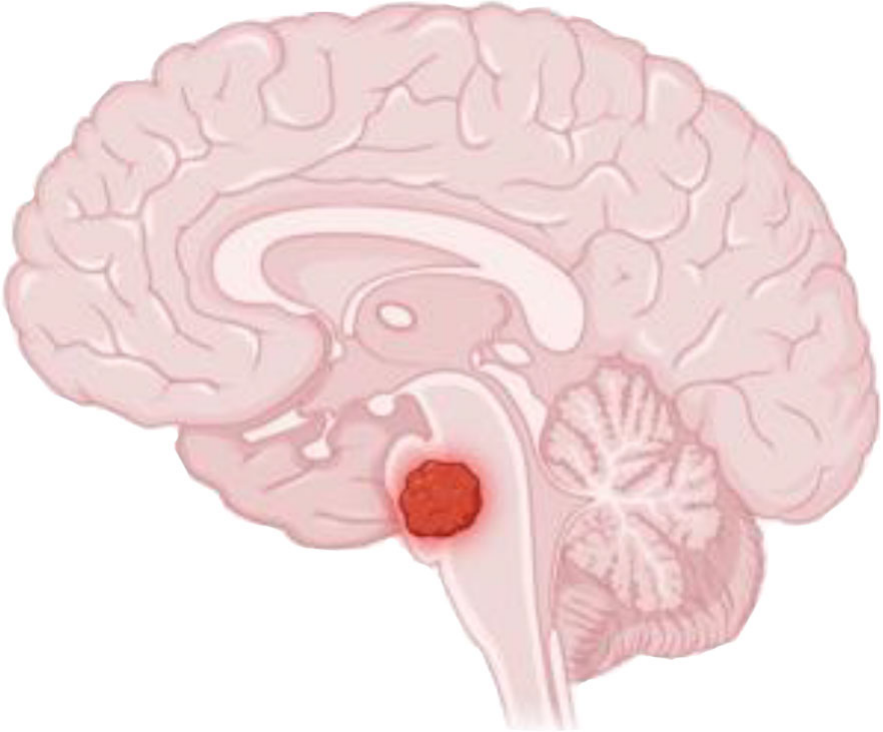	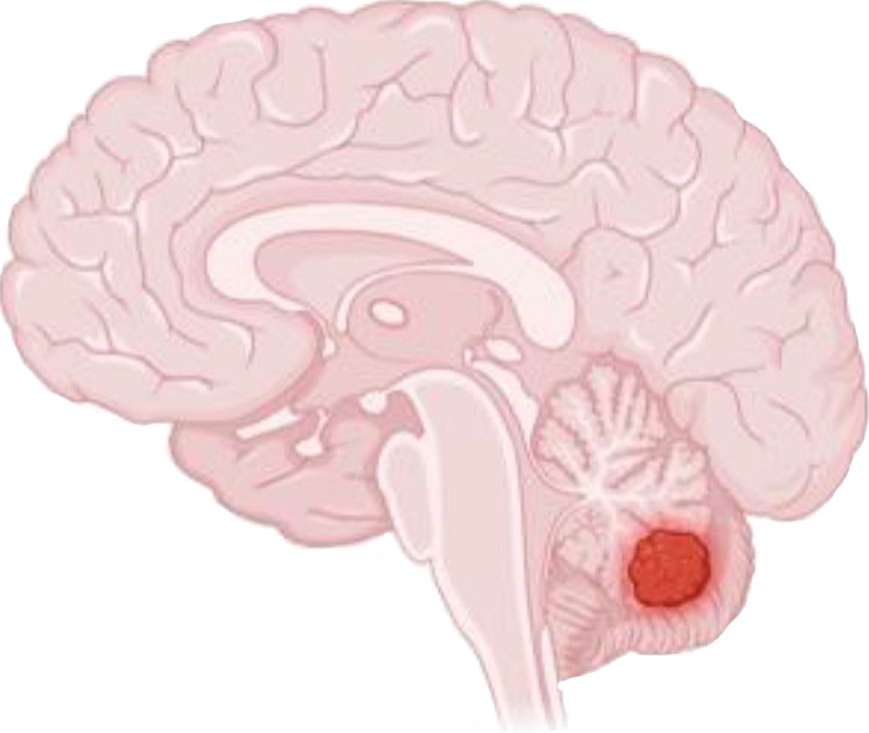
Location	Cerebrum	Pons	Cerebellum
Trafficking Barriers	- Nonuniform BBB disruption - Dense fibrotic extracellular matrices - Irregular vasculature impedes delivery	- Highly intact BBB with limited permeability - Sensitive anatomy limits intervention - Low permeability and tight vasculature	- Variable permeability - Specialized cerebellar vasculature - Anatomical and molecular subtype variability affects infiltration
TME	Highly heterogenous and immunosuppressive	Low immune infiltration and cold microenvironment	Varies by molecular subgroup and can be more immune-permissive
Immune Cells	TAMs, MDSCs, Tregs, microglia and sparse CD8+ T cells	MDSCs, suppressive microglia and few T cell populations	Variable T cell presence, microglia, occasional B cells
Persistence Challenges	- Rapid CAR T cell exhaustion - Heterogeneity promotes antigen escape - Immunosuppressive cytokines dampen effector function	- Lack of costimulatory signals and antigen exposure - Minimal endogenous activity - Low inflammatory signature	- Subtype specific immune evasion mechanisms - Hostile environment and antigen escape due to lack of TME uniformity

Summary of key challenges limiting CAR-T cell trafficking and persistence across glioblastoma (GBM), diffuse intrinsic pontine glioma (DIPG), and medulloblastoma (MB).

**1. *Glioblastoma (GBM):*** GBM, one of the most aggressive and prevalent primary brain tumors, is associated with significant BBB/BTB disruption, particularly in areas exhibiting angiogenesis (the formation of new blood vessels) stimulated by vascular endothelial growth factor (VEGF) (25, 26). In fact, strategies to address the VEGF-induced angiogenic nature of GBM include anti-VEGF agents that can help traffic CAR-T cells to the site of action (27). Nevertheless, the disruption of the BBB/BTB in such regions increases vascular permeability, potentially facilitating the delivery of therapeutic agents, including CAR T cells (28, 29). This may enhance CAR T cell infiltration in specific areas of the tumor (29, 30).

However, the disruption and metamorphosis of the BBB into the BTB is not uniform across the entire tumor. In more aggressive or deeply infiltrative regions of the tumor, the BTB may remain intact, limiting CAR T cell access (4). Furthermore, the immunosuppressive TME and dense extracellular matrices presents additional challenges for CAR T cell function, even in areas where BTB permeability is increased (15, 16). Therefore, while certain areas of GBM may allow CAR T cell penetration, the heterogeneous BBB/BTB disruption complicates comprehensive tumor targeting (31). Another aspect of BTB heterogeneity is the temporal nature of its evolution; the BTB is not static, but changes dynamically, frequently coordinated by tumoral strategies to evade endogenous and exogenous immunological agents (32). This aspect of BTB variability is especially key in aggressive brain malignancies such as GBM (33, 34).

**2. *Diffuse Intrinsic Pontine Glioma (DIPG):*** DIPG, located in the brainstem, presents unique challenges for CAR T cell therapy (35). Unlike GBM, which shows more widespread BTB/BBB disruption, DIPG generally maintains an intact personalized BTB in the brainstem (36). This intact barrier restricts the ability of systemically administered CAR T cells to infiltrate the tumor, as the BTB prevents their entry into the tumor core (35, 37). This impermeability categorizes DIPG as a rather resistant brain tumor as even chemotherapeutic agents fail to achieve effective results (38). The anatomical location of the brainstem also adds another layer of complexity to managing DIPG, as it controls vital functions such as respiration and heart rate (33). Therapies designed to breach the BBB thus risk damaging these critical regions of the brain (38). In addition, even with intrathecal delivery of CAR T cells into cerebrospinal fluid (CSF), the integrity of the BBB in the brainstem remains a major challenge, limiting the effectiveness of the therapy (39).

**3. *Medulloblastoma (MB):*** Medulloblastoma, particularly the aggressive Sonic Hedgehog (SHH), Group 3 and 4 subtypes, typically arises in the cerebellum and presents additional challenges for CAR T cell therapy (40). These tumors often display varying degrees of BTB disruption; the WNT subtype, a relatively less aggressive variant of MB, has a more permeable BTB, whereby, in some regions, compromised BTB integrity may allow for more efficient CAR T cell infiltration (41). However, in the more aggressive MB subtypes, the BTB remains intact, preventing CAR T cells from effectively reaching the tumor (42). Therefore, the prognostic outlook of each MB subtype is different. This is further complicated by discordant respective therapeutic responses to treatment interventions which can variably impart further resistance onto the BTB. Moreover, the anatomical position of the cerebellum within the posterior fossa, and surrounded by specialized vasculature, also complicates therapeutic CAR T cell delivery (43). Therefore, the ability of these immune cells to penetrate the tumor core is restricted by the extent of varying BTB disruption in different areas of MB which constitutes a distinct challenge to therapeutic strategies (41).

**4. *Ependymoma (EPN):*** EPN are rare tumors arising from ependymal cells lining the ventricles or spinal cord; they present specific challenges based on their location within the CNS (44). Ependymomas are typically surrounded by CSF, which further complicates CAR T cell access, particularly if its BTB remains intact in certain regions (45). Furthermore, EPNs located near the brainstem or in the spinal cord may face additional anatomical barriers, such as restricted access to systemic immune therapies (46). Even when BTB disruption occurs, the immunosuppressive TME may further limit CAR T cell efficacy (5, 47). Thus, the nature and behavior of EPN have wide implications onto therapeutic responses to CAR-T cell therapy.

**5. *Atypical Teratoid Rhabdoid Tumor (ATRT):*** ATRT is an aggressive pediatric CNS tumor that often occurs in the posterior fossa, including the cerebellum and brainstem (48). The unique anatomical location of ATRT presents challenges for CAR T cell delivery, as reaching these tumors is dictated by both BBB/BTB integrity and complex vascular structures that limit access of immune cells (49). In cases where ATRT displays significant angiogenesis, certain regions of the tumor may exhibit increased vascular permeability, potentially improving the likelihood of CAR T cell infiltration (50). However, the deep-seated location of these tumors, combined with the potential for elevated intracranial pressure or other delivery challenges, can hinder the effectiveness of CAR T cell therapy (51).

### 3.2 Tumor microenvironment

TMEs play a crucial role in influencing the efficacy of CAR T cell therapy in CNS tumors (5, 52, 53). It encompasses a complex network consisting of cancer cells, stromal cells, immune cells, vasculature, and extracellular matrix (ECM) components, all of which interact to dictate tumor progression and modulate immune responses (54). One of the most significant challenges in CNS tumor therapy is the *heterogeneity* of the TME, which can exist not only between various tumor types but also within different regions of the same tumor; this diversity complicates the development of universal CAR T cell therapies, as the distinct TME characteristics of each tumor can either enhance or obstruct CAR T cell efficacy (52).

1. ***Glioblastoma (GBM):*** GBM is characterized by highly heterogeneous TME: it contains various immunosuppressive factors, including tumor-associated macrophages (TAMs), myeloid-derived suppressor cells (MDSCs), and regulatory T cells (Tregs), which contribute to an environment that dampens T cell activation and function (55). Additionally, the ECM in GBM is often dense and fibrotic, impeding CAR T cell infiltration and migration toward the tumor (56). Hypoxic regions within the tumor core further limit CAR T cell survival and activity (57). Notably, different tumor regions exhibit distinct immune profiles: the tumor periphery may show some signs of immune activation, while the core is more likely to be dominated by suppressive factors such as transforming growth factor-beta (TGF-β), which inhibits CAR T cell function, thus posing a challenge for CAR T cells to uniformly target and eliminate all tumor cells (58).
2. ***Diffuse Intrinsic Pontine Glioma (DIPG):*** DIPG is characterized by an inert or “cold” TME, which subsists of immature blood vessels, a dense ECM, and severe hypoxia, all of which significantly limit the effectiveness of CAR T cell therapies (59). The DIPG TME also contains inhibitory immune cells, such as regulatory T-cells (Tregs) and myeloid-derived suppressive cells (MDSCs), which suppress antitumor immune responses (60). The limited vascularization and resultant hypoxic nature of DIPG hinders CAR T cell infiltration, as the lack of an adequate blood supply prevents efficient delivery of CAR T cells to the tumor site (61). Moreover, the inert TME may impair T cell priming and activation, further limiting CAR T cell efficacy (59). Additionally, DIPG often contains glioma stem cells, which are notoriously resistant to immune attack and can secrete additional immunosuppressive factors that evade immune surveillance (60).
3. ***Medulloblastoma (MB):*** MB is characterized by several subtypes, including WNT, SHH, group 3, and group 4 (62). Each of these subtypes has a heterogeneous TME profile which varies depending on the respective molecular underpinnings (63). For example, Group 3 MB tumors are typically infiltrated by immune-suppressive cells, such as Tregs and MDSCs, which hinder CAR T cell activity (64). Moreover, the ECM in MB can differ in composition: group 4 MB tumors often have a more fibrotic ECM that obstructs CAR T cell infiltration. In contrast, Sonic Hedgehog (SHH)-type MB tumors may have a less immunosuppressive environment but still possess substantial immune evasion mechanisms that prevent effective CAR T cell function (63). A significant challenge in MB is the lack of uniformity in the TME across tumor regions as tumor cells in areas with dense ECM or high levels of immunosuppressive cytokines can effectively shield themselves from CAR T cells, limiting the therapy’s ability to target and eliminate these cells (5, 65).
4. ***Ependymoma (EPN):*** EPN occurs in both the intracranial and spinal cord regions and exhibits TME heterogeneity depending on the tumor location and subtype (66). Intracranial EPN, particularly arising from the ventricular system, tends to have high vascularity but also feature a dense ECM that may obstruct CAR T cell migration (46, 67). These tumors are often associated with elevated levels of TGF-β, interleukin-10 (IL-10), and VEGF, thus creating an immunosuppressive TME that can inhibit CAR T cell activity (68, 69). Spinal EPN, on the other hand, may have a more limited vascular supply, resulting in poorer CAR T cell infiltration (66). The inflammatory cytokines present in the TME can also exacerbate the challenges of CAR T cell penetration, as immune cells such as neutrophils and macrophages may create a hostile environment that hinders CAR T cell engagement and tumor cell killing (66).
5. ***Atypical Teratoid/Rhabdoid Tumor (ATRT):*** ATRT features a heterogeneous TME that also varies by tumor location, as is the case with its other pediatric brain tumor counterparts (69–72). For tumors located in the posterior fossa, including the brainstem, challenges arise due to dense ECM and hypoxic conditions typical of ATRT (57). Like other CNS tumors, ATRT tumors harbor MDSCs, Tregs, and macrophages that contribute to the immunosuppressive microenvironment, actively inhibiting T cell activation and limiting CAR T cell efficacy (73). Additionally, ATRT tumors often exhibit genomic instability, leading to the secretion of immune-modulating factors such as IL-6 and interferons, which may further impair CAR T cell function (74–77).

The heterogeneity of the TME across different CNS tumors thus significantly limits the efficacy of CAR T cell therapy. This is accomplished through several mechanisms:

- ***Immunosuppressive Cells:*** The presence of Tregs, MDSCs, macrophages, and myeloid cells that release immunosuppressive cytokines (e.g., TGF-β, IL-10, IL-6, VEGF) can actively suppress CAR T cell function, diminishing their ability to mount an effective antitumor response (78).
- ***Physical Barriers:*** The dense ECM, fibrosis, and hypoxia within the TME can act as physical barriers that prevent CAR T cells from infiltrating the tumor or migrating to the areas where they are most needed (5). This is particularly evident in tumors with dense stromal components, such as GBM and ATRT (48, 49, 55).
- ***Tumor Heterogeneity:*** Different regions of the same tumor can have varying levels of immune suppression, ECM density, and vascularization.; for example, while one region may exhibit enhanced vascular permeability, another may have an intact BBB and a suppressive immune landscape, complicating the design of universal CAR T cell therapies (1, 40, 52, 79).
- ***Glioma Stem Cells and Immune Evasion:*** Some CNS tumors, such as DIPG and GBM, contain glioma stem cells (GSCs) that are resistant to immune attack (80, 81). These cells secrete factors that enable them to evade immune detection, further hindering the effectiveness of CAR T cells (5).
- ***Altered Cytokine and Growth Factor Production:*** Tumors such as EPN and ATRT may produce altered levels of cytokines and growth factors that shift the TME toward an environment that actively resists CAR T cell action (82). These factors can attract suppressive immune cells or directly interfere with CAR T cell activity (83).

Therefore, the heterogeneity of the TME across different CNS tumor types is a critical factor that limits the efficacy of CAR T cell therapy. Variations in immune cell populations, ECM composition, cytokine profiles, and vascularization create both physical and immunosuppressive barriers to CAR T cell function (39, 52). Moreover, the diversity in TME characteristics complicates the development of therapies that can effectively target and eliminate tumors across these complex and varied microenvironments (5, 54). Addressing these architectural and TME-related challenges will be essential for improving the therapeutic potential of CAR T cells in CNS tumors.

### 3.3 CAR T cell retention and persistence in CNS tumors

The persistence and retention of CAR T cells within the CNS are critical factors in determining the therapeutic efficacy of CAR T cell therapy for CNS tumors (84). Sustained CAR T cell activity and their ability to continuously target tumor cells are essential for achieving durable tumor control and preventing recurrence (85). However, clinical studies have consistently highlighted suboptimal CAR T cell persistence, often associated with tumor relapses or disease progression; this challenge is largely attributed to the immune-privileged nature of the brain and spinal cord, as well as the immunosuppressive and complex characteristics of the TME (63, 86). For instance, in trials investigating CAR-T cells in GBM, which included anti-GD2 (NCT04196413) and anti-EGFRvIII (NCT02209376) CARs, detection was only sustained at the post-infusion stage, thus exhibiting short-lived persistence, with exhaustion and loss of function contributing to tumor recurrence (37, 87). Similarly, in DIPG trials (NCT03500991 and NCT03618381) targeting HER2, GD2, and EGFR, CAR T cells were initially detected following infusion, but persistence was limited, particularly in brainstem tumors (88, 89). Factors such as the intact or partially disrupted BBB and the highly immunosuppressive TME restricted their long-term activity with limited adoptive T cell durability and tumor progression (21, 24, 30). Moreover, a CAR-T trial involving MB, which investigated GD2 CAR T cells (NCT04099797), demonstrated detectable activity in early phases but showed poor long-term persistence, especially in Group 3 MB, where the TME is dominated by immunosuppressive elements (90, 91). Trials for CAR-T cell interventions in ATRT and EPN are limited given the rarity of these malignancies, but preclinical studies and anecdotal data suggest that CAR T cells are transiently detectable post-infusion despite experiencing rapid attrition due to immune evasive mechanisms and the dense ECM further exacerbating CAR-T cell persistence (48, 66, 69, 92, 93).

Pre-clinical and clinical experiences thus highlight that the challenges related to CAR T cell persistence stem from several complex and interrelated factors, including T cell exhaustion, immune evasion mechanisms within the TME, the presence of physical barriers like the BBB, and the immunosuppressive cytokine milieu characteristic of various CNS tumors. These variables are summarized below:

*a.* ***Immunosuppressive TME:*** As detailed in the previous section, the immunosuppressive TME in many CNS tumors presents a significant barrier to CAR T cell persistence (84). Tumors such as GBM, DIPG, and MB often contain suppressive immune cells, including Tregs, MDSCs, and macrophages, which not only limit CAR T cell efficacy but also drive T cell exhaustion (94). Additionally, the secretion of immune-suppressive cytokines such as TGF-β, IL-10, and IL-6 actively inhibits CAR T cell expansion and persistence, reducing long-term survival of therapeutic T cells at the tumor site (86, 95, 96).

*b.* ***Immune Privilege and BBB:*** The CNS is considered an immune-privileged site largely due to the restrictive nature of the BBB, which limits the entry of immune cells, including CAR T cells, from the systemic circulation into the brain and spinal cord (21–23, 30, 97). This limited and often delayed infiltration hinders the persistence of adoptively transferred T cells at the tumor site (4). While some CNS tumors may partially disrupt the BBB, as evidenced by detectable CAR T cell infiltration following systemic administration, the BBB and the blood-tumor barrier (BTB) remain significant physical obstacles which impede uniform CAR T cell access to the tumor, particularly to its core, thus challenging CAR-T cell retention at the site of action, ultimately restricting sustained CAR T cell activity within the tumor microenvironment (98).

*c.* ***Tumor Heterogeneity:*** Tumors such as GBM, MB, and DIPG exhibit intra-tumoral heterogeneity, where different regions of the tumor may possess varying immune cell populations, vascularization, and hypoxic zones (99). This heterogeneity creates regions where CAR T cell infiltration is severely limited. Even in areas where CAR T cells successfully penetrate, they often become rapidly exhausted after initial activity, compromising their persistence and cytotoxic function across the entire tumor mass (100) Moreover, tumors can adapt to immune pressure by altering antigen expression or upregulating immunosuppressive ligands, further impeding CAR T cell survival and efficacy within the TME (101).

In addition to these challenges, antigen heterogeneity across CNS tumors presents a major barrier to effective CAR T cell therapy. Targeting a single antigen or epitope often fails to eradicate all tumor cells due to variable levels of antigen expression within and between tumors (15, 93, 102). In some cases, subpopulations of tumor cells may completely lack expression of the targeted antigen, enabling immune escape even in the presence of active CAR T cells. Additionally, tumors may express truncated or mutated forms of antigens that are not recognized by the CAR, further diminishing therapeutic efficacy. Antigen downregulation following selective immune pressure contributes to inconsistent therapeutic outcomes and tumor relapses. For instance, in GBM, heterogeneous expression of antigens such as EGFRvIII has led to incomplete tumor targeting and subsequent relapses after EGFRvIII-directed CAR T cell therapy. Similarly, in medulloblastoma, the differential antigenic expression of markers such as HER2 or B7-H3 across tumor subpopulations creates reservoirs of antigen-negative cells that can evade immune clearance. These issues are especially problematic in solid tumors, which tend to exhibit greater genetic instability and more hostile microenvironments compared to hematological malignancies, where malignant cells generally maintain more stable antigen profiles (103).

*d.* ***CAR T Cell Exhaustion and Phenotypic Changes:* CAR** T cells frequently experience exhaustion due to sustained exposure to tumor antigens and immune-suppressive factors within the TME (94). This exhaustion is characterized by the upregulation of inhibitory receptors, such as programmed death-1 (PD-1), T cell immunoglobulin and mucin-domain-containing protein 3 (TIM-3), and lymphocyte activation gene 3 (LAG-3), which diminish CAR T cell functionality and persistence (104). Additionally, epigenetic remodeling plays a significant role in driving exhaustion by inducing stable, heritable changes in gene expression that reinforce the exhausted phenotype (105). Furthermore, CAR T cells often differentiate into terminally differentiated subsets, such as effector T cells, or into less effective memory subsets, both of which exhibit reduced proliferative capacity and impaired long-term antitumor activity (106). These phenotypic and epigenetic changes collectively undermine CAR T cell persistence and therapeutic efficacy in CNS tumors (84, 95).

*e.* ***Cytokine Environment and Interleukin (IL) Signaling:*** The cytokine milieu in the CNS TME plays a pivotal role in determining the survival and persistence of CAR T cells (107). In CNS tumors, high levels of IL-10, TGF-β, and IL-6 are often secreted, contributing to immune suppression and CAR T cell attrition (108). The lack of supportive cytokines, such as IL-2, which promotes T cell survival, can further contribute to the reduced persistence of CAR T cells in the CNS, leading to an early termination of the therapeutic response (109).

Therefore, the limited persistence of CAR T cells in CNS tumors remains a major barrier to successful clinical outcomes (110). Factors such as immunosuppressive mechanisms in the TME, physical barriers like the BBB, and tumor heterogeneity contribute to the early exhaustion and loss of CAR T cells, leading to disease progression or tumor relapse (111). Overcoming these challenges requires a deeper understanding of how the TME impacts CAR T cell engraftment and persistence, as well as the development of strategies to enhance CAR T cell survival and functionality in the CNS.

## 4 Strategies for overcoming current challenges

Several pre-clinical studies and clinical trials have focused on potential strategies to improve CAR T cell design, manufacturing processes, and combination therapies, as well as incorporating additional engineering and genetic modifications to overcome challenges related to persistence and exhaustion (112–114). In this section, we will break down these approaches and provide additional perspectives on novel potential solutions to address these critical obstacles (**Table 3**).

**Table 3:** Summary of challenges and respective solutions to CAR-T cell therapy in the context of malignant primary brain tumors.

Challenge	Innovative Solutions
Enhancing Brain Infiltration of CAR-T Cells	1.Advanced Delivery Strategies: • Direct tumor site injection (locoregional) • Refined systemic administration techniques • Novel intranasal delivery methods
	2. Cutting-edge Delivery Technologies: •Nanoparticle and exosome-based transport systems • Engineered biomaterials for targeted cell delivery • BBB modulation (pharmacological or radiological interventions)
Improving CAR-T Cell Longevity and Function	1.Enhanced Cell Manufacturing: • Selective T cell subpopulation harvesting • Optimized cell culture protocols
	2. Advanced CAR Engineering: • Next-generation receptor designs • Strategic gene editing (e.g., CRISPR-based modifications)
	3. Multi-pronged Targeting Approaches: • Dual-antigen recognition (tandem CARs) • Multiple CAR expression systems • Conditional activation CAR designs (logic-gated)
	4. In-situ CAR-T Generation: • Direct in-vivo T cell genetic modification
	5. Synergistic Therapy Combinations: • Cytokine-secreting "armored" CAR-T cells • CAR-T cells engineered to produce antibodies • Coordinated administration of immunomodulators
	6. Tumor Microenvironment Modulation (e.g., FUS-assisted CAR-T infiltration and activation)

### 4.1 Mechanisms to overcome trafficking hurdles

#### 4.1.1 Modulating method of CAR-T delivery

**Figure 4:**
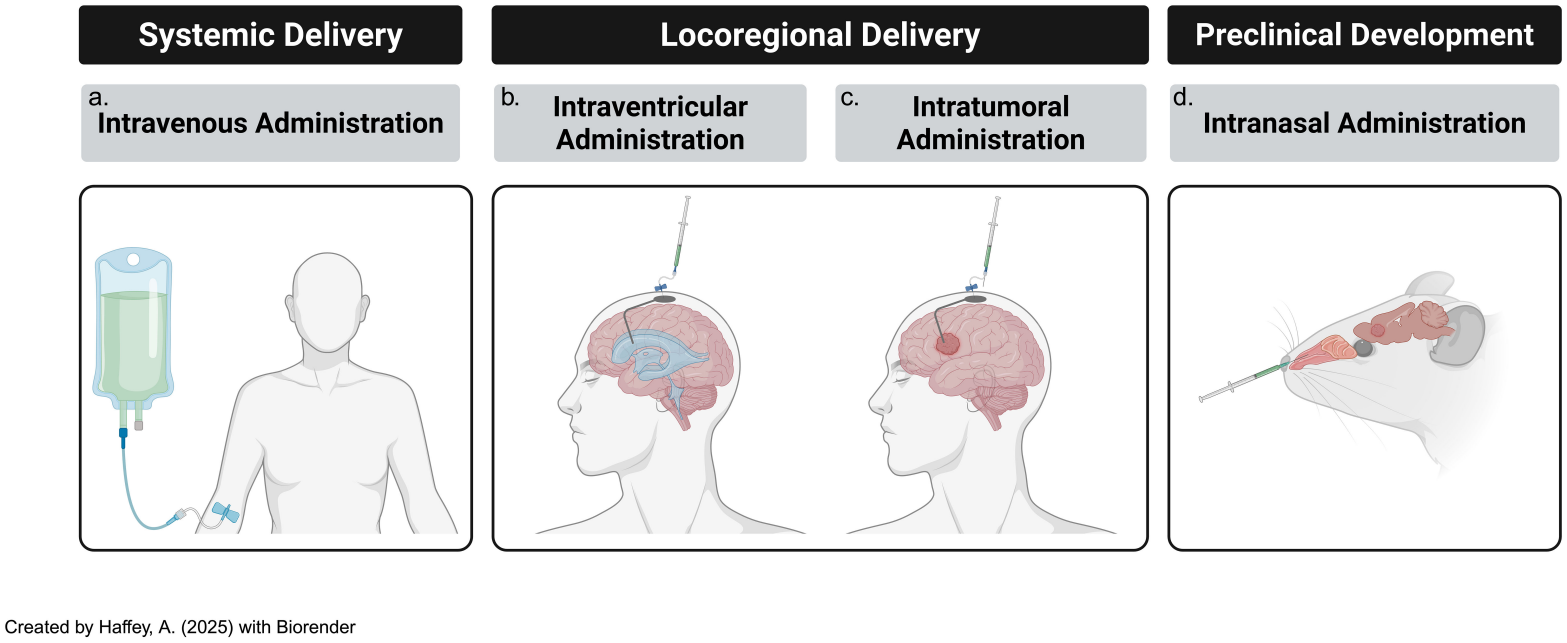
Modes of delivery of adoptive t-cells, including CAR-Ts, subsists of several tested paths [including **(a)** systemic & **(b)** locoregional routes] and **(c)** novel, developing techniques such as intranasal routes of administration. All three modes of delivery are consistently updated with novel iterations that attempt to ameliorate previous challenges..

Optimizing the route of CAR-T cell administration is a critical factor in enhancing therapeutic efficacy for CNS tumors; both locoregional and systemic injection modalities have been investigated, each with distinct advantages and limitations depending on tumor characteristics and the physiological barriers present in the CNS (**Figure 4**) (115).

**Locoregional Administration**: Intraventricular and intrathecal routes of CAR-T cell delivery have shown promise in preclinical models for targeting CNS tumors; for instance, intraventricular CAR-T cell infusion in group 3 MB mouse models resulted in significantly improved therapeutic outcomes and reduced systemic toxicity compared to intravenous delivery (116). The key benefit of locoregional administration is its ability to bypass the BBB, allowing direct delivery of CAR-T cells to the tumor site, thus minimizing off-target effects and systemic adverse events (117). However, its major limitation lies in restricted spatial targeting, which may not effectively address disseminated disease or micro metastases (118, 119). Additionally, given their invasive nature, procedures like intraventricular cannula implantation or repeated intrathecal injections may pose challenges for long-term patient management.

**Systemic Administration**: Intravenous infusion of CAR-T cells provides the advantage of widespread distribution, potentially reaching distant tumor sites and micro-metastases that locoregional approaches may miss; however, the BBB remains a major hurdle in ensuring that CAR-T cells reach brain tumors (4). Despite this challenge, systemic delivery offers several benefits, including simpler administration in outpatient settings, reduced reliance on invasive procedures, and greater likelihood of treating diffuse or residual tumors that are less amenable to locoregional targeting (120).

**Intranasal Delivery**: Another emerging approach is intranasal administration of CAR-T cells, which constitutes a direct pathway to the brain, thereby bypassing the BBB to a certain extent, and facilitating greater infiltration of CAR-T cells into the brain to treat malignant or autoimmune diseases (121). This approach can minimize risk of systemic exposure due to direct brain access but also has potential for more feasible maintenance therapy convenience, in addition to enhanced patient comfort and compliance.

While preclinical studies such as those incorporating HER-2 targeting CAR-T cells and non-engineered cytotoxic lymphocytes (CTLs) in murine GBM models have demonstrated that intranasal delivery of immune cells can lead to migration within brain regions affected by disease, this method is still in its early stages, and requires rigorous testing to ensure safety and efficacy, particularly given the heterogeneous nature of BBB disruption across various CNS tumor variants (21).

#### 4.1.2 Leveraging novel delivery platforms

**Leveraging Nanocarrier Systems**: Nanotechnology presents a promising strategy for enhancing the delivery of CAR-T cell therapy, particularly in overcoming the challenges of the blood-brain barrier (BBB) and improving tumor targeting (122). By encapsulating CAR-T cells or related therapeutic agents in nanoparticles, it is possible to enhance BBB penetration and deliver the cells more efficiently to tumor sites (123–125). Nanoparticles can also carry immunomodulatory agents, such as RNA vaccines or immune checkpoint inhibitors, which can improve CAR-T cell function and persistence within the tumor microenvironment (126–128). Additionally, magnetic nanoparticles have been studied in combination with CAR-T cells, demonstrating reduced toxicity and improved tumor targeting when CAR-T cells are attached to the nanoparticles (129).

A form of nanocarrier technology includes exosomes, which are naturally occurring *extracellular vesicles (EVs)* secreted by cells containing a multitude of molecular mediators unique to their source of production (130). EV research in the field of cancer therapeutics has yielded a plethora of data points delineating the potential of their use in diseases such as Non-Hodgkin Lymphoma (NHL) as diagnostic, prognostic, and therapeutic tools (131). The latter includes incorporating Chimeric Antigen Receptors; studies have revealed various promising but precarious therapeutic endpoints of combining EVs harboring targets like those of CAR-T cells. Nevertheless, they have shown promise as *substitutes* to CAR-T cells given the possibility of loading EVs with CARs on their surface to target and avert tumor growth (131). Notwithstanding these innovative strategies, EVs themselves harbor potential for ameliorating CAR-T cell therapy, whereby cellular CAR constructs or other acellular therapeutic agents are delivered directly to brain tumors via EVs (132). Exosomes have also been shown to cross the BBB, making them an effective vehicle for delivering CAR-T cell–derived therapeutic cargo in a controlled and targeted manner (133). One key advantage of using exosomes is their ability to modulate the tumor microenvironment (TME), which can enhance CAR-T cell efficacy (134). By utilizing these innovative delivery methods, it may be possible to improve CAR-T cell therapy outcomes and reduce off-target effects, ultimately enhancing the therapeutic potential for CNS tumors (130). Moreover, they can be loaded with surface markers that serve as CAR-T “backpacks” to delivery therapeutic payloads (e.g., loaded with cytokines) that coincide with CAR-T cell action at the tumor site (131). Furthermore, interventions such as chemically grafting molecular moieties like short-chain fatty acids (SCFAs) and tryptophan metabolites, which are microbial products that have a specialized traversal paths across the BBB, onto payload nanocarriers such as EVs, can forge the way forward towards more innovative strategies of ameliorating difficulties associated with delivering CAR-T-supportive agents to the CNS (135).

While each method incorporating nanocarrier modalities to improve immunotherapeutic strategies and attenuate obstacles offers unique benefits, their effectiveness will ultimately depend on addressing the complexities of tumor microenvironments and BBB permeability (99, 134). Continued innovation and further research in this field could substantially improve the outcomes of CAR-T cell therapy for brain tumors.

**Utilizing Biomaterial Formulations for CAR-T Cell Delivery:** Biomaterials present an innovative approach to enhancing CAR-T cell therapy by providing a platform for delivering CAR-T cells and supporting adjunct agents, thus improving their persistence and function within the tumor microenvironment (TME) (136–138). Hydrogel-based microstructures have been explored as scaffolds for co-delivering therapeutic agents, including small molecules (e.g., chemotherapeutic drugs) and cytokines (e.g., IL-15) to enhance CAR-T cell activity (139–141). These hydrogels are biocompatible, biodegradable, and capable of controlled release, allowing for sustained delivery of agents directly at the tumor site (142). In preclinical studies, CAR-T cells targeting melanoma marker chondroitin sulfate proteoglycan 4 (CSPG4) were combined with hydrogels containing IL-15 and platelets (141). The addition of cytokines in the hydrogel formulation improved CAR-T cell persistence and proliferation, potentially overcoming the challenges imposed by the TME (141).

Hydrogel microstructures are also beneficial for bypassing dense tumor barriers, facilitating better CAR-T cell infiltration and expansion within the tumor (143). These innovations are particularly valuable for treating brain tumors, where the TME is characterized by barriers such as the BBB and a dense extracellular matrix (ECM) that restrict drug and cell delivery (144). The clinical feasibility of hydrogel-based formulations is dependent on their safe implantation and compatibility with patient-specific tumor characteristics. For example, a collagenase nanogel biomaterial equipped with CXCR4 antagonism was shown to efficiently help traffic CAR-T cells to the site of pancreatic solid tumors in a pancreatic tumor model through overcoming traditional ECM (extracellular matrix) barriers, thus facilitating a “CAR-T/collagenase nanogel backpack” delivery system (145). Furthermore, fibrin-based hydrogels have been tested in glioblastoma models and were shown to improve CAR-T cell outcomes compared to direct injections (146). Fibrin’s biocompatibility, along with its ability to support CAR-T cell growth and modify the immunosuppressive TME, makes it a promising candidate for clinical application, especially in pediatric brain tumors where the BBB and dense ECM create significant challenges for drug and cell delivery (147). Therefore, further incorporating biomaterial usage in the realm of brain tumor therapeutics may be key in overcoming key challenges in immunotherapeutic strategy implementation.

**Chemical Manipulation of the BBB:** As previously noted, the BBB is a formidable barrier to therapeutic delivery, including CAR-T cells (23). Various methods have thus been explored to transiently disrupt the BBB to improve the delivery of therapeutic agents. Strategies such as intra-arterial administration of mannitol have been frequently attempted, but with notable adverse effects. More recently, agents like xNEO100 (a purified form of P-OH, or perillyl alcohol) have shown promise in enhancing BBB permeability (148). This agent has been investigated *in-vivo* for treating GBM and demonstrated the ability to reversibly permeabilize the BBB by loosening endothelial tight junction proteins (149). Compared to mannitol, NEO100 is more efficient, requiring lower concentrations and achieving better BBB disruption in a dose- and time-dependent manner (150). One clinical implication of using NEO100 is its potential combination with CAR-T cell therapy to improve targeting and immune cell recruitment, such as CD8+ T cells, within the brain tumor microenvironment (151). In addition, mouse models have shown that co-administration of NEO100 with PD-1 inhibitors and CAR-T cells enhances both delivery and therapeutic efficacy (148). Such combination therapies could improve CAR-T cell localization to brain tumors and boost the overall immune response, leading to better patient outcomes; however, careful monitoring of systemic toxicity, particularly in combination with immune checkpoint inhibitors, is essential to ensuring patient safety (152).

**Figure 5:**
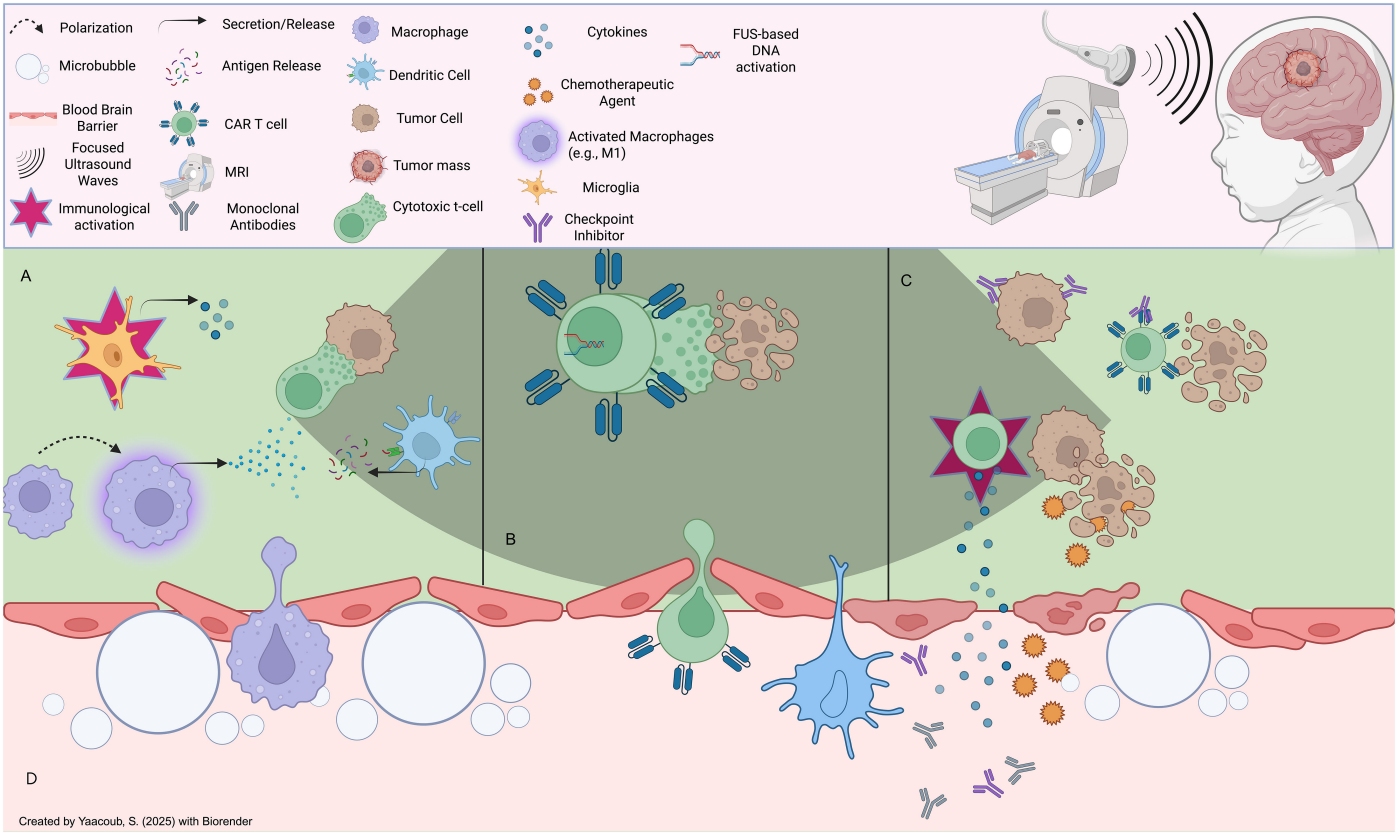
Effects of focused ultrasound on the tumor microenvironment: **(A)** FUS induces immunomodulatory effects onto immune cells in the TME, such as microglial activation, macrophage repolarization (e.g., M2 to M1), and antigenic presentation of tumor neoantigens onto immune cells (eg., T cells), **(B)** FUS can be leveraged to induce acousto-genetic, controlled CAR-T cell activation to limit unwanted pro-inflammatory effects (e.g., adverse effects onto host tissues) **(C)** FUS-mediated BBB opening can mediate CAR-T cell infiltration post-systemic administration intravenously, in addition to entry of other therapeutic agents such as endogenous or extrinsic cytokine and antibody-based therapies that can induce anti-tumor effects. **(D)** FUS induces reversible BBB disruption through microbubble-based cavitation effects which can loosen tight junctions; this can lead to the infiltration of a plethora of endogenous immune cells as well as extemal therapeutic agents.

**Applying Focused Ultrasound (FUS):** FUS is a non-invasive, non-ionizing technique that transiently opens the BBB, facilitating improved delivery of therapeutic agents, including CAR-T cells, to brain tumors (153). This process is enhanced through the introduction of microbubbles (µB) that, when exposed to acoustic waves, create mechanical oscillations that disrupt the tight junctions of the BBB, allowing for deeper penetration of therapeutic agents (**Figure 5**) (154, 155). Clinical trials have demonstrated the potential of FUS in various brain tumor types, including GBM, DIPG, and brain metastases (156–162). For instance, at the University of Maryland Medical Center, MRI-guided FUS was used to open the BBB and improve the delivery of chemotherapeutic agents to GBM (163), while in DIPG, FUS enhanced the delivery of doxorubicin (164). Additionally, FUS has been shown to increase the penetration of trastuzumab into the brain in patients with brain metastasis (165). Early clinical trials combining FUS with CAR-T cells for pediatric patients with aggressive brain tumors such as DIPG and MB have shown encouraging results, with improved delivery of CAR-T cells and other therapeutic agents like immune checkpoint inhibitors to the tumor site (155, 166–169). This approach has the potential to increase survival rates and enhance CAR-T cell therapy efficacy in pediatric populations. However, challenges persist, such as optimizing treatment parameters (e.g., sonication intensity and duration) to minimize tissue damage. Moreover, evaluating the long-term effects of repeated FUS treatments on brain tissue will be essential in optimizing parameters (166). Despite these challenges, FUS has significant potential to revolutionize CAR-T cell therapy for brain tumors by improving BBB permeability and ensuring that CAR-T cells can effectively reach and treat tumors within the brain.

Therefore, overcoming the trafficking hurdles associated with CAR-T cell therapy for CNS tumors involves optimizing injection modalities, leveraging nanotechnology and exosomes, and employing both chemical and physical techniques to disrupt the BBB. These strategies present promising opportunities to enhance the efficacy of CAR-T cell therapy. Ongoing research and innovation in these areas are essential for improving treatment outcomes for patients with brain tumors.

### 4.2 Strategies to enhance CAR-T cell persistence

**Figure 6:**
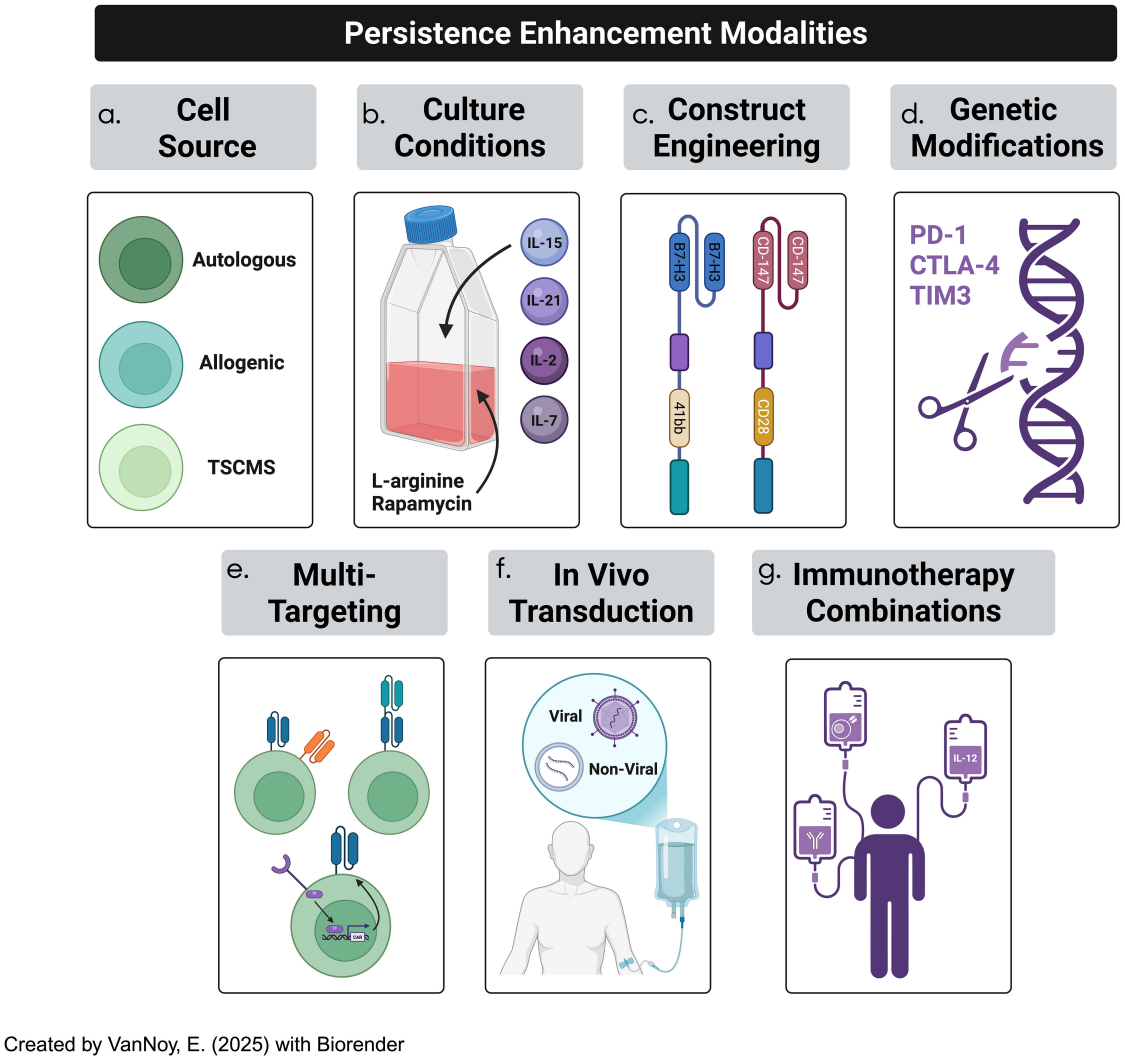
CAR-T cell therapy efficacy in solid tumor treatment has been often hindered by lack of persistence due to a plethora of factors. Several strategies to overcome persistence challenges occurs through **(a)** cell-source optimization, **(b)** culturing conditions, **(c)** optimizing CAR constructs, **(d)** genetic modification of CAR constructs, **(e)** adopting multi-targeted techniques, **(f)** employing in-vivo T cell transduction into CAR-T cells, and **(g)** incorporating immunotherapeutic combinations.

The long-term effectiveness of CAR-T cell therapy relies on the persistence and functionality of engineered T cells at the tumor site (113). However, CAR-T cells often struggle to maintain activity, particularly within an immunosuppressive TME (52). To overcome these challenges, strategies have been developed to enhance CAR-T cell persistence, including optimizing the T cell source, refining cell culture conditions, modifying CAR constructs, and utilizing genetic engineering techniques (**Figure 6**). The overarching nuances in CAR-T cell synthesis in the traditional pipeline include, but are not limited to, patient-specific variability, the phenotypes of T cell subsets derived from peculiar sources (autologous vs other), the transduction success of CAR constructs, culturing conditions, and CAR construct designs (113). Moreover, on a larger scale, the feasibility of administering CAR-T cells to patient populations requires near-perfect standards; this includes product consistency, cryopreservation impact, product turnaround time, Good Manufacturing Practice (GMP) standards, and scalability and cost implications. For the scope of this manuscript, we will focus on a handful of all these variables.

#### 4.2.1 T cell source optimization

The persistence of CAR-T cells is influenced by their origin, which can be either autologous (derived from the patient’s own T cells) or allogeneic (sourced from a healthy donor) (86). *Autologous* CAR-T cells are favored in most clinical settings due to their lower risk of immune rejection and graft-versus-host disease (GvHD), but they are often associated with reduced long-term persistence in the tumor microenvironment (170). This persistence is hindered by factors such as T cell exhaustion and tonic signaling (94, 171). Additionally, the logistical complexities and high costs associated with autologous therapies present significant challenges (172). *Allogeneic* CAR-T cells, on the other hand, offer advantages such as cost-efficiency and scalable manufacturing, as they can be pre-sourced and stored for use in multiple patients; however, the risk of GvHD and immune rejection due to the HLA mismatch remains a key concern (173, 174). Advances in genetic engineering, including the knockout of T-cell receptors (TCRs) or HLA modification, are being explored to mitigate these risks, although such modifications must be carefully optimized to minimize off-target effects (175). Recent advancements have also highlighted the potential of using stem memory T-cells (TSCMs), a rare but highly durable subset of T cells that exhibit self-renewal capacity and can persist long-term; these T cells are less prone to differentiation and exhaustion compared to conventional T cells, making them ideal candidates for CAR-T cell therapies targeting solid tumors (176).

#### 4.2.2 Optimizing T cell culture conditions

Cell culture conditions play a pivotal role in determining the functionality, expansion, and persistence of CAR-T cells (177). During the ex-vivo expansion phase, the cytokine milieu and culture environment both influence the differentiation and longevity of the engineered T cells (109). Traditional expansion protocols often rely on IL-2 for cell proliferation; however, IL-2 promotes differentiation into effector T cells, which are typically less persistent and more prone to exhaustion (177). Recent studies have suggested that the use of IL-7, IL-15, and IL-21 in culture can promote a less differentiated and longer-lasting phenotype, characterized by a stem cell memory phenotype that enhances both persistence and anti-tumor efficacy (178, 179). These cytokines are more effective at maintaining a population of central memory T-cells (TCM) and stem cell memory T-cells (TSCM), which are associated with improved long-term survival and robust anti-tumor responses (179). Additionally, metabolic programming has emerged as a critical aspect of CAR-T cell persistence: T cells undergoing aerobic glycolysis (the Warburg effect) tend to differentiate into effector cells, which are short-lived and less effective (180). By promoting oxidative phosphorylation and inhibiting glycolysis, the metabolic reprogramming of T cells can support a memory-like phenotype and enhance their durability (181). In particular, the use of mTOR inhibitors like rapamycin and L-arginine supplementation has been shown to improve metabolic fitness and support long-term persistence (182).

#### 4.2.3 Engineering CAR constructs for enhanced persistence

The design of the CAR construct itself is critical to enhancing T cell persistence; the extracellular binding domain, transmembrane domain, and intracellular signaling domains all play pivotal roles in shaping the function and longevity of CAR-T cells (17). The single-chain variable fragment (scFv) dictates antigen specificity and binding affinity, both of which have considerable impact on tonic signaling and desired specific targeting (17). The hinge and transmembrane domains also have pivotal implications on how the CAR-T engages antigens such that T-cell fitness and epitope targeting is heavily influenced (183). Moreover, the selection of intracellular binding domains, including the co-stimulatory and primary activation domains, has been shown to alter the metrics of CAR-T cell effectiveness; this is embodied by improved short-term efficacy, offset by diminished persistence, and vice-versa (183). Finally, CAR constructs have been developed over the years such that various (five) generations exist, each of which has driven different groups to study their relative effectiveness.

Notably, the inclusion of co-stimulatory signaling domains such as 4-1BB and CD28 has profound implications for T cell persistence (184). While CD28 provides rapid expansion and potent short-term effector functions, it can also induce early exhaustion; in contrast, 4-1BB-based CARs promote a more sustained T cell activation and a memory phenotype, which is associated with improved long-term persistence and anti-tumor efficacy (185). Further optimization of the CAR construct can reduce the likelihood of tonic signaling, which can lead to premature activation and exhaustion of CAR-T cells (86, 96). The design of the scFv (single-chain variable fragment), including the spacer and hinge regions, must be carefully adjusted to prevent tonic signaling without compromising CAR-T cell activation (186). Moreover, incorporating transient CAR expression systems, where CAR expression is controlled based on external signals, could provide a more balanced activation response and prevent premature exhaustion (187).

#### 4.2.4 Genetic modifications to enhance CAR-T cell longevity

The non-durable effects of CAR-T cells in CNS tumor treatment have led to increasing interest in genetic modifications of CAR-T cells to improve their longevity and functional persistence (188). One promising approach is the genetic knockout of immune checkpoints such as PD-1, CTLA-4, and TIM-3; these inhibitory receptors are generally upregulated in response to chronic antigen exposure, leading to T cell exhaustion (189). By knocking out or inhibiting these molecules, researchers can potentiate CAR-T cell function and enhance persistence (113). CRISPR/Cas9 technology has enabled precise, targeted gene editing in CAR-T cells, allowing for the deletion of specific genes involved in immune regulation (190). For example, knocking out PD-1 has shown promise in preclinical glioma studies by improving CAR-T cell activity, particularly in immune-suppressive tumor microenvironments (191). However, the risk of off-target effects and unintended mutations necessitates careful monitoring and validation before these approaches can be translated into clinical practice. Additionally, another emerging strategy is the manipulation of DNA methylation pathways to modulate T cell exhaustion; for instance, the reduction of DNMT3a expression, a key DNA methyltransferase, has been shown to sustain a relatively undifferentiated phenotype in CAR-T cells, leading to better persistence and anti-tumor responses (192). This modification, coupled with epigenetic reprogramming, offers a novel way to prolong the effectiveness of CAR-T therapies.

#### 4.2.5 Multi-targeting approaches

To address the challenges of tumor heterogeneity and antigen escape in brain tumors, multi-targeting strategies are emerging as a promising approach in CAR-T cell therapy (193). These approaches aim to enhance the specificity and efficacy of CAR-T cells by enabling them to recognize and target multiple tumor-associated antigens simultaneously. By diversifying the targets, multi-targeting strategies reduce the risk of antigen escape and improve the likelihood of complete tumor eradication, especially in heterogeneous tumor populations like those found in CNS malignancies (194).

- **Tandem CARs**: These combine two or more CAR constructs into a single molecule, allowing simultaneous targeting of multiple antigens, thus increasing the likelihood of tumor elimination, even in cases of antigen escape (195).
- **Dual and Multi-CAR Constructs**: Dual CARs express two distinct CARs targeting different antigens, while multi-CAR constructs target several antigens (196). These strategies broaden the range of tumor recognition, enhancing efficacy and preventing relapses.
- **Synthetic Notch (synNotch) CARs**: SynNotch CARs enable conditional activation based on antigen recognition, reducing off-tumor toxicity and ensuring T cell activation only in the presence of specific antigens, enhancing safety and specificity (197, 198).
- **Co-activation Systems**: These systems require the recognition of two or more antigens for CAR-T cells to be activated, ensuring precise targeting of tumor cells while minimizing damage to healthy tissues (199).
- **Logic-Gated CARs**: These integrate multiple antigen signals, activating CAR-T cells only when specific combinations are recognized, improving control over T cell activation and reducing off-tumor effects (200).
- **Split-CARs**: Split-CARs require the presence of two separate components expressed on different T cells, ensuring activation only when both signals converge at the tumor site, and reducing off-target toxicity (201).

Incorporating these multi-targeting designs into CAR-T cell therapies offers a promising strategy to improve efficacy and safety, addressing key obstacles in treating CNS tumors. In summary, the enhancement of CAR-T cell persistence requires a comprehensive approach that includes optimizing the T cell source, refining culture conditions, engineering CAR constructs, and incorporating genetic modifications. By addressing these factors, CAR-T cell therapies can be made more durable and effective, particularly for treating challenging pediatric brain tumors, where sustained persistence is essential for achieving long-term therapeutic success.

#### 4.2.6 *In vivo* CAR-T cell transduction

*In vivo* CAR-T cell transduction represents an innovative strategy to enhance CAR-T cell therapy for CNS tumors (202). This strategy remains in a relatively infantile stage of development, but has great implications on manufacturing bottlenecks, scalability/accessibility concerns, and alleviated obstacles such as potential for shorter window times of repeated dosing and rapid treatment indices. This method modifies T cells directly within the body, avoiding the need for ex vivo expansion (203). Techniques such as viral vectors (lentivirus, adenovirus) or non-viral methods like CRISPR-Cas9 and mRNA-based systems can be used to introduce CAR constructs into T cells circulating in the bloodstream or within the tumor microenvironment (204). This approach has the potential to simplify CAR-T cell production and reduce the time required for therapy. *In vivo* transduction may also enable better control over the persistence and expansion of CAR-T cells within the body, enhancing the durability of therapeutic responses (205).

#### 4.2.7 Implementing immunotherapy combinations

Advances in CAR-T cell therapy underscore the importance of synergistic combinations to enhance the persistence, expansion, and efficacy of CAR-T cells (206). Combining CAR-T cells with pharmacological agents or radiological techniques that improve cell trafficking, enhance penetration, or mitigate the effects of the immunosuppressive TME presents a promising strategy for improving therapeutic outcomes (207, 208).

##### 4.2.7.1 CAR-T and monoclonal antibodies

Combining CAR-T cell therapy with monoclonal antibodies targeting immune checkpoints offers a promising strategy to overcome the challenges posed by immune evasion mechanisms (189). Key immune checkpoints, including PD-1, PD-L1, CTLA-4, TIM-3, TIGIT, and LAG-3, are critical regulators of T-cell exhaustion and immune suppression, making them valuable targets for combination therapies (209, 210). Checkpoint inhibitors such as PD-1 and PD-L1 blockers (nivolumab, pembrolizumab) and CTLA-4 inhibitors (ipilimumab) have shown clinical success in a range of cancers; combining these agents with CAR-T cells aims to relieve T-cell exhaustion, enhance anti-tumor immunity, and improve CAR-T cell persistence within the TME (209, 210). Preclinical studies have shown that combining CAR-T cells targeting EGFRvIII with anti-PD-1 or anti-PD-L1 antibodies results in significant tumor regression in GBM models, indicating that checkpoint blockade can potentiate CAR-T cell activity (211). Additionally, emerging studies investigating the role of targeted anti-CD47 agents in oncological diseases constitute valuable attempts at inducing immunomodulatory changes capable of potentiating accompanying immunotherapeutic strategies such as CAR-T therapies (212). However, safety concerns associated with systemic administration of checkpoint inhibitors and other monoclonal antibody agents, which have been shown to occasionally lead to significant toxicity and undesirable immunological adverse sequelae, are paramount, which underscores the need for additional investigations and an open mind to alternative approaches (213).

One promising strategy is engineering CAR-T cells to express checkpoint inhibitors on their surface, thereby overcoming the limitations of systemic delivery (214). CAR-T cells engineered to secrete PD-1 blocking antibodies have demonstrated improved anti-tumor efficacy and reduced exhaustion in solid tumor models, including glioblastoma; this approach not only enhances CAR-T cell proliferation and cytotoxicity but also ensures sustained activity within the immunosuppressive TME (215). In addition to PD-1 and PD-L1, other immune checkpoints like TIGIT and LAG-3 are gaining attention for their role in immune suppression (216). TIGIT inhibits T-cell activation by binding to its ligand, CD155, while LAG-3 downregulates T-cell function through its interaction with MHC class II molecules (217). Combining blockade of TIGIT and PD-1, or TIGIT and LAG-3, has been shown to improve CAR-T cell efficacy in preclinical studies of solid tumors, supporting the use of combination strategies that target multiple immune checkpoints (218). Furthermore, antibodies targeting other immune-regulatory pathways, such as the inhibitory receptor TIM-3 or the co-inhibitory molecule VISTA, are being explored in combination with CAR-T therapy (218). These molecules are involved in regulating T-cell activity in tumors and could provide additional avenues to enhance CAR-T cell persistence and efficacy.

Overall, the integration of checkpoint inhibitors, both through systemic administration and engineering CAR-T cells to express them, represents an exciting direction for improving the persistence, expansion, and therapeutic efficacy of CAR-T cells in treating CNS tumors and other solid cancers. Continued research into other immune-regulatory pathways will likely lead to more refined combination strategies, further advancing CAR-T cell therapy for challenging malignancies.

##### 4.2.7.2 CAR-T and cytokine modulation

Cytokine support plays a crucial role in the activation, proliferation, and long-term persistence of CAR-T cells; cytokines IL-2, IL-15, IL-12, and others have been extensively studied for their ability to modulate immune responses and enhance the therapeutic efficacy of CAR-T cells (219, 220). While cytokines can significantly improve CAR-T cell function, their systemic administration often comes with significant risks, including toxicity (221). As a result, innovative approaches are being developed to localize cytokine delivery to the TME, minimizing systemic side effects while maximizing therapeutic benefits (221).

**IL-2 and IL-15: Expanding and Sustaining CAR-T Cells:** IL-2 has long been used to promote T-cell expansion due to its potent stimulatory effects; however, its use is often limited by the risk of systemic toxicity, including vascular leak syndrome, which can lead to serious complications (222). To overcome this challenge, researchers have focused on engineering “armored” CAR-T cells capable of secreting cytokines locally within the TME (223). This localized delivery helps modulate the immune response at the tumor site while avoiding systemic side effects. Although IL-2 remains valuable for expanding CAR-T cells, alternatives like IL-15 are becoming increasingly popular for their ability to promote CAR-T cell expansion and persistence without the same level of toxicity (224). IL-15 is particularly promising because it supports long-lasting T-cell immunity, improves T-cell generation, and enhances the durability of CAR-T cell therapies (224). The incorporation of IL-15 or IL-15 super agonists in CAR-T cell constructs has shown great promise in preclinical models, particularly for solid tumors, where the TME often limits the persistence and effectiveness of CAR-T cells (225).

**IL-12: Modulating the TME for Improved CAR-T Function:** IL-12 has garnered attention as a potent immune-modulatory cytokine that enhances anti-tumor immunity by promoting the differentiation of Th1 cells and activating macrophages, dendritic cells, and NK cells; importantly, it also plays a critical role in driving a pro-inflammatory TME, which is favorable for CAR-T cell function (226). In preclinical models of glioblastoma, the combination of CAR-T cells with IL-12 demonstrated improved tumor infiltration, reduced PD-1 expression on T cells, and enhanced anti-tumor cytotoxicity (227). Importantly, IL-12 has been shown to induce a TME more conducive to CAR-T cell survival, thus addressing one of the major challenges in solid tumors—immune suppression (228). By secreting IL-12 locally, CAR-T cells can enhance their anti-tumor effects, reduce T-cell exhaustion, and further improve persistence within the hostile TME of CNS tumors (229).

**T-Cell Engagers: Targeting Cytokines and Cytokine Receptors:** Beyond traditional cytokine supplementation, recent studies have explored the development of T-cell engagers that combine CAR-T cells with co-expressed cytokines or cytokine receptors. These innovative designs enable CAR-T cells to release cytokines in a controlled, localized manner within the TME, enhancing their activation and persistence without triggering widespread systemic effects (230). For instance, CAR-T cells engineered to express a high-affinity IL-2 receptor (CD25) can leverage the use of IL-2 present in the TME, leading to sustained expansion and prolonged persistence of CAR-T cells at the tumor site. This localized approach to cytokine delivery has the potential to overcome one of the key barriers in CAR-T cell therapy for solid tumors—persistence.

As the understanding of the TME evolves, the development of more sophisticated cytokine-based strategies will be essential for advancing CAR-T cell therapies. Targeted modulation of the TME through cytokines will likely become a cornerstone of combination therapy approaches, enhancing the immune response and ultimately improving patient outcomes, particularly for pediatric brain cancers and other challenging solid tumors.

##### 4.2.7.3 CAR T cells and FUS

As introduced in previous sections, FUS is an innovative technique that temporarily disrupts the BBB, significantly improving the delivery of CAR-T cells to the tumor site (231). By overcoming the BBB, FUS facilitates CAR-T cell infiltration into CNS tumors (232, 233). Beyond aiding CAR-T cell penetration, FUS also plays a crucial role in modulating the TME through inducing sterile inflammation, recruiting immune cells, and activating microglia, all of which enhance the anti-tumor immune response (234) (**Figure 6**). When combined with checkpoint inhibitors, such as anti-PD-1, FUS helps reverse immune suppression within the TME, further boosting CAR-T cell efficacy (157, 166). Studies have shown that this combination significantly improves CAR-T cell activity and enhances tumor control (168). Moreover, FUS can disrupt the tumor’s ECM, through reducing tumor compactness via loosening the dense structure of the ECM, thus improving access for CAR-T cells (235). The enhanced tissue permeability following FUS treatment is thus crucial for improving CAR-T cell delivery and overall therapeutic efficacy.

Another promising development involves combining FUS with gene-modified CAR-T cells engineered to respond to FUS-induced activation (187). This strategy enables more localized and controlled activation of CAR-T cells at the tumor site, minimizing systemic toxicity and enhancing their therapeutic potential (187). Preclinical studies have demonstrated that this combination not only improves tumor control but also extends survival, offering a novel approach to treating brain tumors and other solid cancers (236). FUS can also be combined with other immunomodulatory agents or therapeutic compounds to further enhance CAR-T cell activity (156, 231).

The synergy between CAR-T cells and FUS addresses the critical challenge of BBB penetration while enhancing CAR-T cell access and infiltration by targeting the ECM. By combining these innovative approaches, this strategy holds significant promise for improving the treatment of brain tumors, offering new hope for patients with these challenging cancers.

##### 4.2.7.4 CAR T cells and other emerging therapeutic modalities

Other emerging therapeutic modalities also have the potential to transform the landscape of CAR-T cell therapy:

**Gene Editing for TME Modulation:** Advances in gene editing technologies such as CRISPR-Cas9 allow for precise modifications of the CAR-T cell genome to improve functionality in solid tumors (188). CAR-T cells can be edited to knock out genes that promote immune suppression, such as PD-1, or to express pro-inflammatory cytokines and chemokines that recruit other immune cells to the tumor site (214, 237, 238). In addition, gene editing can be used to manipulate the TME directly, improving tumor responsiveness to CAR-T cells (239).

**Oncolytic Viruses:** Oncolytic viruses (OVs) that selectively infect and kill tumor cells while stimulating anti-tumor immunity are emerging as powerful adjuncts to CAR-T cell therapy (240). These viruses can enhance CAR-T cell infiltration into tumors, modulate the TME, and induce immune responses (241). When used in combination, OVs can synergize with CAR-T cells by triggering tumor cell death, releasing tumor antigens, and promoting a more immunostimulatory environment (242).

**Radiation Therapy:** While traditionally used as a standalone modality, radiation therapy can enhance CAR-T cell efficacy by inducing immunogenic cell death and promoting antigen release (243). Combining low-dose radiation with CAR-T therapy has been shown to enhance CAR-T cell activity in solid tumors by inducing favorable changes in the TME, such as upregulation of tumor-associated antigens and increased immune cell infiltration (244).

**CAR T Cell Manufacturing:** Innovations in automated and closed-system platforms have streamlined CAR-T cell production, improving scalability, reducing costs, and minimizing contamination risks (245). Advances in viral transduction, including lentiviral and retroviral vector engineering, have improved gene transfer efficiency, reduced production times, and enhanced safety profiles (246). Additionally, next-generation CAR-T cells with enhanced functionality and allogeneic “off-the-shelf” therapies are accelerating clinical applications, making CAR-T therapy more accessible and efficient (103). Adopting immunotherapy combinations involving CAR-T cell therapy with monoclonal antibodies, cytokines, FUS, and other innovative therapies therefore holds great promise for overcoming the limitations of CAR-T cells in treating solid tumors, particularly in the context of the immunosuppressive brain tumor microenvironment. These combination strategies aim to enhance CAR-T cell persistence, modulate the TME, and potentiate anti-tumor immunity, potentially leading to improved therapeutic outcomes for patients with brain malignancies. However, clinical translation of these combination therapies will require rigorous evaluation of safety, efficacy, and potential long-term side effects in human patients.

## 5 Future perspectives

### 5.1 Tracking CAR-T cells

CAR-T cell therapy has revolutionized cancer treatment, especially for hematologic malignancies, but its translation to brain tumors and other solid cancers presents unique challenges. In this paper, we outlined a plethora of possible solutions that can be put forth to ameliorate obstacles in the face of CAR-T cell therapy in pediatric brain tumors (**Table 4**). One of the critical barriers is the lack of straightforward biomarkers to monitor CAR-T cells, unlike traditional chemical agents where serum concentration is often used as an indicator of efficacy and toxicity (247). Moreover, the personalized nature of autologous CAR-T cell therapy results in heterogeneous responses, making it challenging to predict therapeutic effects and manage potential toxicities (119). Consequently, there is a growing need for effective, non-invasive techniques to track CAR-T cells *in vivo* and evaluate their therapeutic impact.

**Table 4:** Current therapeutic strategies to enhance CAR T Cell efficacy: An overview of emerging therapeutic strategies and their current stage of development aimed at overcoming challenges in treating pediatric brain tumors.

Strategy	Purpose	Translational stage status	Lead example	Publications/Clinical trials
Intraventricular Delivery 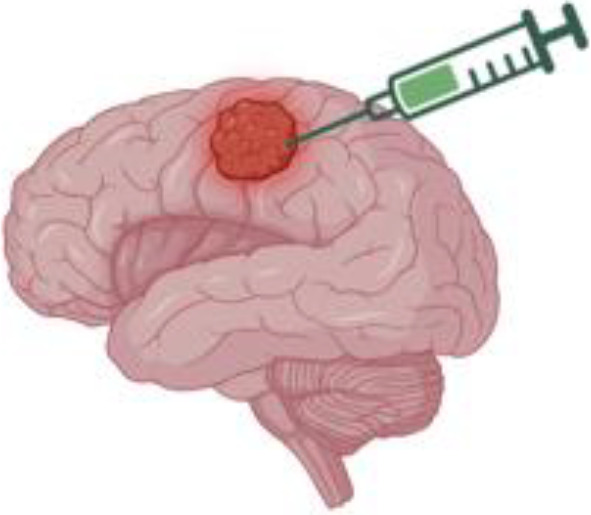	Direct administration to bypass BBB	Active clinical trials for GBM + DIPG, Preclinical for MB	Intracranial GD2 CAR T cells for H3K27M+ diffuse midline glioma	NCT03638167 NCT04196413 Donovan et al., 2020 (116)
Nanotechnology 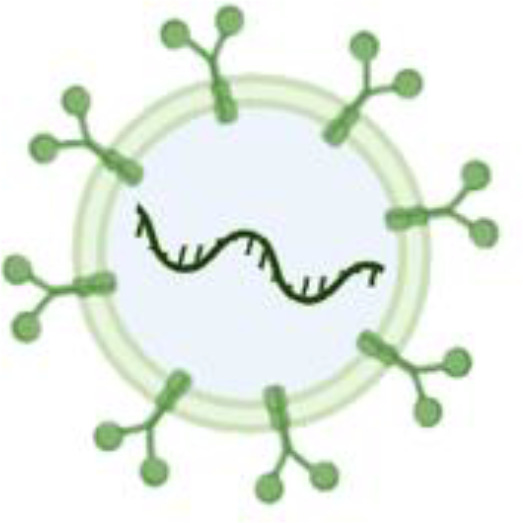	Enhance BBB penetration and payload delivery to tumor sites	Advanced and exploratory preclinical	Loading of CAR-T cells with magnetic nanoparticles for controlled targeting	Pfister et al., 2025 (129) Chang et al., 2023 (124) Sani et al., 2024 (133)
Biomaterials 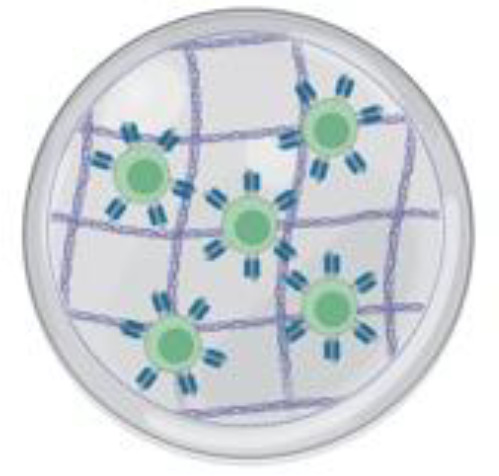	Enhance local CAR-T delivery and survival within tumor bed	Preclinical for GBM	Fibrin gel enhances anti-glioma effects of CAR T cells	Ogunnaike et al., 2021 (146) Mellati et al., 2021 (139) Nguyen et al., 2023 (137)
BBB Chemical Manipulation 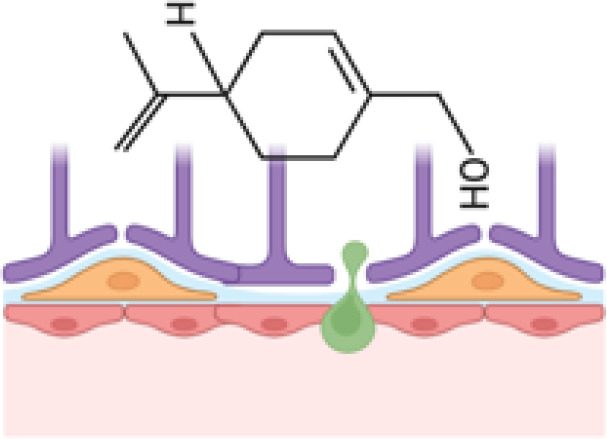	Improve delivery of therapeutic agents	Preclinical in combination with CAR T	Co-administration of NEO100 and CAR T	Wang et al., 2021 Wang et al., 2024 (151)
Focused Ultrasound 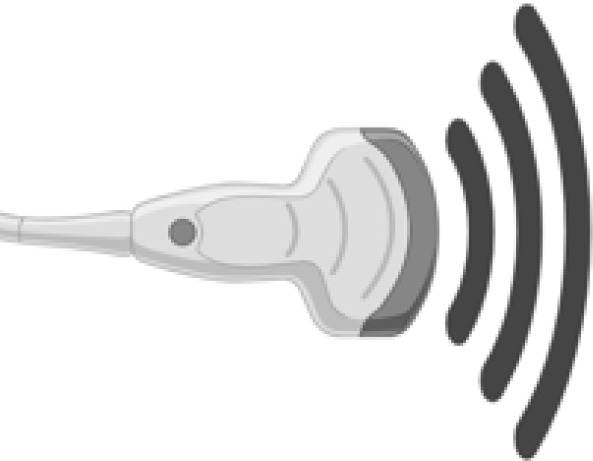	Transient opening of BBB to deliver therapeutic agents	Preclinical for CAR T delivery, early-stage clinical trials for chemotherapeutic agents and checkpoint inhibitors	FUS enhanced delivery of doxorubicin in gliomas	NCT06329570 NCT04116320 Sabbagh et al., 2021 (155) Arrieta et al., 2024 (164)
Genetic Modifications 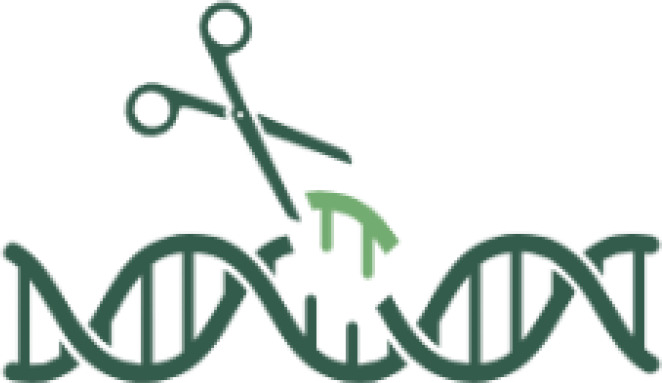	Improved longevity and persistence of CAR T cells	Mostly preclinical, CRISPR CARs in early clinical trials for other cancers	PD1 disruption enhances CAR T efficacy	NCT03545815 Rupp et al., 2017 (190) Prinzing et al., 2021 (192)
Multi-Targeting 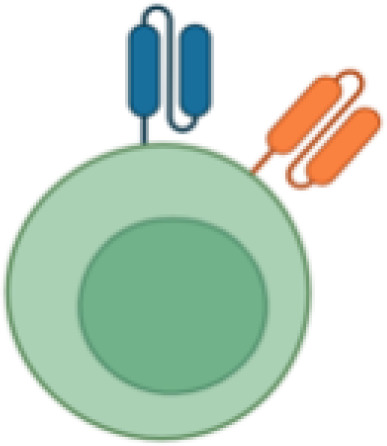	To address tumor heterogeneity and antigen escape	Active clinical trials for GBM, DIPG and MB	Tandem EGFRvIII and IL-13Ra2 targeting against heterogenous GBM	NCT05168423 NCT05768880 Schmidts, et al 2023 (195)
Combination Therapy 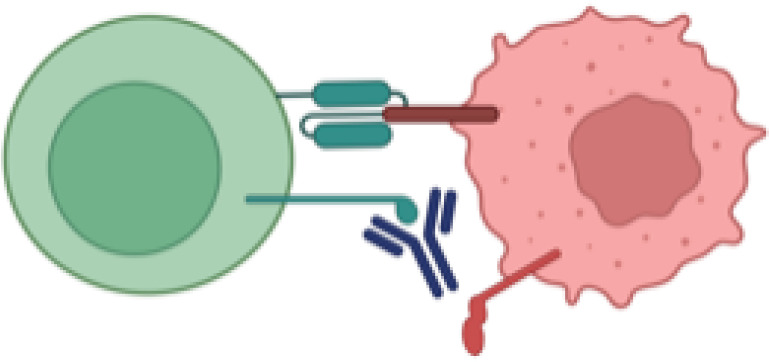	Synergistic combinations to enhance efficacy of CAR T cells	Active clinical trials for GBM, DIPG and MB	EGFRvIII CAR T combined with Pembrolizumab in GBM	NCT04099797 NCT03726515 NCT04003649
Cytokine Armoring 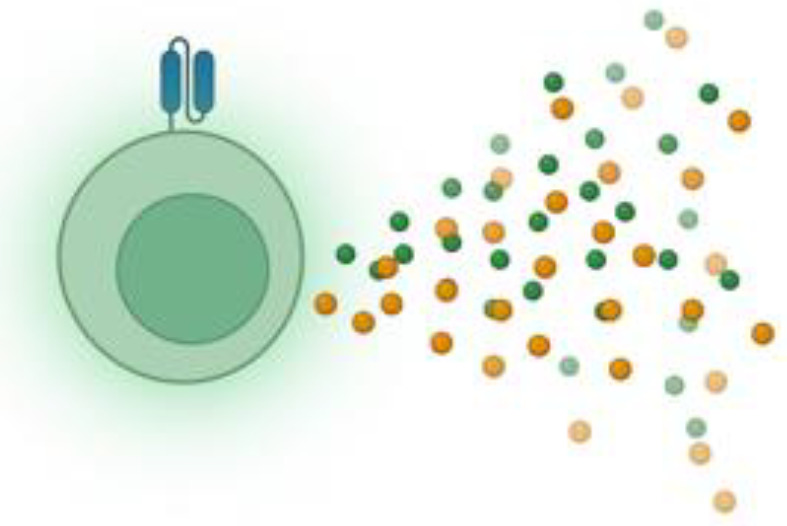	Local cytokine secretion in the TME	Predominantly preclinical	IL15 enhances CAR T cell function in GBM	Brog et al., 2022 (223) Alizadeh et al., 2019 (224)

Non-invasive imaging techniques have therefore emerged as a promising solution for real-time monitoring of CAR-T cells (248). These techniques involve either direct labeling of CAR-T cells (ex-vivo marking and subsequent labeling) or indirect labeling, where the cells are genetically modified to express a reporter gene (249). Direct labeling, while useful in some cases, faces limitations such as dilution during cell division, reducing the tracking efficacy over time (250). This issue complicates long-term monitoring, particularly in clinical settings. On the other hand, indirect labeling, which involves incorporating radiolabeled reporter genes into CAR-T cells, enables more efficient tracking (249). This method allows monitoring of cell expansion over time, although it remains technically challenging for routine clinical application.

In addition to traditional labeling methods, alternative technologies such as nanoparticle-based techniques and microfluidic platforms are being explored to enhance the efficiency and precision of CAR-T cell tracking (251, 252). These techniques promise to overcome the limitations of current labeling methods by providing higher sensitivity and more accurate imaging, facilitating the assessment of CAR-T cell migration, distribution, and functional patterns (252). As research progresses, these imaging techniques will likely become invaluable for evaluating the *in vivo* behavior of CAR-T cells, improving patient-specific treatment strategies and optimizing therapy outcomes.

Various imaging modalities have also been explored for monitoring labeled CAR-T cells. Common techniques include CT, PET, and PET/CT, which are well-established for assessing tumor responses (253). For CAR-T cell imaging, specific criteria must be met to ensure the successful translation of these methods into clinical practice. The reporter molecules used in these imaging systems must be highly specific to the CAR-T cells, non-immunogenic, and sensitive enough to detect low quantities of the cells (254). Additionally, these reporters should have minimal expression profiles in normal tissues to reduce background noise and allow precise tracking of CAR-T cells, especially when administered to the brain (255). The ability of these reporters to cross the BBB and not alter the CAR-T cells’ functionality is also a critical consideration. Therefore, clinical success of these imaging strategies will depend on the integration of these criteria, influencing how CAR-T cell therapies are monitored in clinical trials and how toxicity and efficacy are evaluated in brain tumor models.

### 5.2 Toxicities of CAR T-cell therapy in CNS tumors

CAR T-cell therapy has shown transformative potential in treating various malignancies, including CNS tumors. However, its clinical application, particularly in brain tumors, is accompanied by significant toxicities that require careful management (256, 257). These toxicities, primarily associated with cytokine release syndrome (CRS) and immune effector cell-associated neurotoxicity syndrome (ICANS), pose unique challenges due to the delicate and enclosed nature of the CNS (258).

**CRS:** Cytokine release syndrome is a systemic inflammatory response triggered by CAR T-cell activation and proliferation, which releases large amounts of cytokines (259). CRS has been well documented as a dose-limiting toxicity in hematological cancers, but its manifestation in CNS tumors differs (260). In CNS solid tumors, the onset of CRS is typically delayed, occurring as CAR T cells gradually infiltrate the tumor and undergo full activation (256). This delayed onset contrasts with the more immediate inflammatory response seen in hematological malignancies, where tumor cells are disseminated throughout the body, leading to quicker systemic inflammation (260).

While CRS is less frequent and less severe in patients with solid tumors, including CNS malignancies, it remains a significant concern. The compromised vasculature of brain tumors, which is impacted by the BBB/BTB, limits CAR T-cell infiltration and may slow down the onset of CRS (261, 262). However, when CRS does occur, it can still be severe, requiring immediate intervention (256). The typical clinical management strategies for CRS include the administration of tocilizumab, an IL-6 receptor inhibitor, which has shown efficacy in mitigating cytokine release without significantly impairing CAR T-cell activity. In cases of severe CRS, corticosteroids may also be employed, although their use can reduce CAR T-cell effectiveness and inhibit tumor clearance (260).

Recent studies suggest that the use of pre-treatment cytokine levels as biomarkers could help predict the likelihood of CRS and guide the management of patients at higher risk (263). This approach could improve patient stratification and allow for more personalized treatment regimens, minimizing the incidence of severe CRS.

**ICANS:** A critical and unique toxicity in the context of CAR T-cell therapy for CNS tumors is ICANS (256). ICANS is characterized by a range of neurological symptoms, including encephalopathy, cerebral edema, seizures, and aphasia (264). The CNS-specific nature of this toxicity presents unique clinical challenges, as it can be difficult to distinguish between tumor progression and the onset of ICANS, especially in patients with brain tumors. This diagnostic challenge complicates the management of these patients, as physicians must differentiate between true tumor growth and neurotoxic effects induced by CAR T-cell treatment.

ICANS occurs because of CAR T-cell infiltration into the brain, where localized inflammation causes neurotoxicity (264, 265). Localized delivery of CAR T-cells, such as through intracranial or intraventricular infusion, is being explored as a strategy to mitigate systemic side effects and reduce the risk of ICANS (117, 266). However, this approach does not eliminate the risk of neurotoxic effects. Indeed, even localized CAR T-cell therapy can lead to severe neurotoxicity in some patients, particularly if the CAR T-cells trigger an intense inflammatory response within the confined environment of the CNS (265).

Management of ICANS typically involves the use of corticosteroids to suppress inflammation; however, corticosteroid use can impair CAR T-cell functionality, presenting a difficult balance between managing toxicity and maintaining therapeutic efficacy (267). Tocilizumab, commonly used to manage CRS, is also occasionally used to address ICANS, but its effectiveness in the context of neurotoxicity is still under investigation (257). In addition to corticosteroids and IL-6 inhibitors, emerging strategies are being explored to mitigate neurotoxicity without compromising CAR T-cell activity. These include modifying CAR T-cell constructs to reduce the release of pro-inflammatory cytokines, as well as incorporating specific immunosuppressive agents that target neuroinflammation while preserving CAR T-cell function (257, 258).

**Clinical Management Strategies and Emerging Approaches:** The clinical management of CAR T-cell therapy toxicities in CNS tumors is evolving as the field advances. In addition to corticosteroids and tocilizumab, other therapies, such as Janus kinase (JAK) inhibitors, are being explored to manage cytokine-driven inflammation with potentially fewer impacts on CAR T-cell efficacy (268). Moreover, pre-treatment screening for biomarkers that predict the risk of CRS and ICANS is an active area of research (265). Several studies have indicated that baseline levels of cytokines such as IL-6 and IL-1, as well as tumor burden and CAR T-cell expansion kinetics, may serve as predictive markers for adverse events (263, 265).

In the realm of CAR T-cell engineering, significant progress is being made to minimize toxicities while enhancing therapeutic efficacy (269). Advances in novel CAR design modifications, such as dual-target CAR T-cells, are designed to target both the tumor and the immune microenvironment, show promise in reducing off-target effects and improving specificity (269). Additionally, research into “safety switches” that allow for controlled elimination of CAR T-cells in the event of severe toxicities is gaining traction to mitigate risks associated with both CRS and ICANS (267, 270).

### 5.3 Current clinical landscape and translational bottlenecks in CAR T cell therapy for CNS tumors

Significant strides have been made in advancing CAR T cell therapy for CNS tumors, with early clinical trials demonstrating feasibility and initial safety. However, major biological, regulatory, and logistical challenges continue to hinder the broad and effective clinical implementation of these therapies.

In **Table 1**, we summarize a selection of notable early-phase clinical trials (primarily Phase 1) evaluating CAR T cell therapy in CNS tumors. These include trials targeting GD2 (e.g., NCT04196413, NCT04099797), HER2 (NCT03500991, NCT03596073), B7-H3 (NCT04815307), and IL13Rα2 (NCT02208362). Across these trials, common primary endpoints include safety, tolerability, and dose-limiting toxicities, while secondary endpoints involve CAR T cell persistence, tumor response by imaging (RANO criteria), and survival outcomes.

Although these trials confirm that intracranial and locoregional delivery of CAR T cells is generally safe and feasible, clinical outcomes remain modest. For instance:

- In NCT02208362, IL13Rα2 CAR T cells delivered intratumorally in GBM showed transient radiographic responses, but tumor regrowth occurred within months, and CAR T cells were no longer detectable after peak expansion.
- GD2-targeting trials (NCT04196413) in diffuse midline glioma demonstrated the ability of CAR T cells to traffic to CSF and tumor sites, yet persistence remained limited and correlated with transient responses.
- In NCT03500991, HER2 CAR T cells infused intracranially in pediatric CNS tumors showed no dose-limiting toxicities, but antitumor activity was minimal, highlighting challenges in potency and durability.

These findings collectively underscore key biological limitations, including suboptimal trafficking, low CAR T cell persistence, and limited immune activation in the hostile CNS tumor microenvironment.

Beyond biologic barriers, translational and regulatory bottlenecks also constrain progress:

- Autologous manufacturing is slow, often taking 3–4 weeks. This is unfeasible for rapidly progressing CNS malignancies like glioblastoma or DIPG.
- Product heterogeneity (e.g., variable transduction efficiency, inconsistent memory phenotypes) introduces uncertainty in clinical outcomes.
- There is a lack of standardized management protocols for treatment-related neurotoxicity, especially ICANS, which may manifest differently in patients with preexisting CNS pathology.
- Preclinical models are frequently immunodeficient and do not recapitulate human tumor–immune interactions, hampering predictive accuracy of efficacy and safety.
- GMP and regulatory hurdles, including requirements for clean-room manufacturing, release testing, and quality control, create bottlenecks, particularly in resource-limited settings or academic institutions.
- Accessibility and equity issues remain, as most trials are conducted at specialized centers, leaving patients in underserved regions with limited options.

Regulatory challenges in pediatric CAR T trials are significant due to stricter safety requirements, longer follow-up mandates, and the need for age-specific dosing and neurodevelopmental risk assessments. These factors often delay trial approval and limit patient enrollment. Given the rarity of pediatric CNS tumors, trials tend to be small and underpowered. Streamlining regulatory processes and creating pediatric-focused development frameworks will be key to accelerating safe and effective CAR T therapies for children.

To overcome these barriers, a multi-pronged and coordinated global approach is needed. Strategies may include the development of allogeneic or off-the-shelf CAR T products, decentralized manufacturing platforms, improvement in preclinical immunocompetent CNS tumor models, and harmonization of regulatory pathways to reduce delays while maintaining safety standards. Integration of biomarker-driven adaptive trials and real-time translational feedback loops can further accelerate refinement and deployment of effective CAR T cell therapies for CNS tumors.

### 5.4 Clinical translation

CAR T-cell therapy holds tremendous potential in revolutionizing cancer treatment, especially for CNS solid tumors that have historically shown minimal to no long-term responses to traditional therapies. The transition of various CAR T-cell models from preclinical studies to clinical application, however, faces significant challenges that require a multifaceted approach.

One of the major obstacles to successful clinical translation is the failure of preclinical models to adequately replicate the TME and immune system dynamics of humans. Non-syngeneic (non-immunocompetent) murine models, which are commonly used in preclinical research, are limited in their ability to mimic the complexity of human immune interactions. These models fall short in accounting for the full spectrum of immune mediators, immune checkpoints, and cytokine environments that influence CAR T-cell behavior in humans. While syngeneic (immunocompetent) murine models are more effective in providing a closer approximation of human immune responses, they present their own challenges. Particularly developing CAR T-cell therapies targeting tumor-specific antigens that are not expressed on normal host tissues thus requires careful optimization to avoid potential off-target effects.

Moreover, translating CAR T-cell therapies from animal models to human patients involves addressing several other critical considerations, such as optimizing CAR construct design, improving T-cell manufacturing processes, and overcoming regulatory hurdles. The combination of mono- or combination therapies, which may target multiple antigens or modulate the immune microenvironment, holds promise for improving the efficacy of CAR T-cells in solid CNS tumors. However, ensuring that these therapies are both effective and safe will require extensive research into the identification of appropriate antigens, the design of CAR constructs with improved specificity, and the development of scalable and standardized protocols for T-cell production.

In summary, while CAR T-cell therapy represents a promising breakthrough for treating resistant CNS solid tumors, achieving successful clinical translation demands concerted efforts to refine preclinical models, improve CAR T-cell engineering, and address logistical and regulatory challenges. These combined efforts will be essential in making CAR T-cell therapy a viable, standardized treatment option for CNS cancers.

## 6 Discussion and conclusion

As it stands, the lack of success of CAR T cell therapy in brain malignancies highlights the urgent need for innovative therapeutic strategies. However, the unique challenges of CNS tumors complicate this translation. The relevant variables include inadequate tumor infiltration, immune suppression within the TME, the restrictive nature of the BBB, and systemic toxicities associated with immune activation. Overcoming these obstacles requires a multidisciplinary approach that integrates biological insights with cutting-edge engineering advancements.

A range of innovative solutions has emerged to address these barriers. Locoregional delivery systems, such as intra-tumoral injections, micro-injectable hydrogels, and implantable scaffolds, can facilitate direct CAR-T cell administration to tumor sites while minimizing systemic exposure. Moreover, the integration of FUS acts as a promising modality for temporarily disrupting the BBB and subsequently enhancing CAR-T cell delivery to CNS tumors. Beyond improving trafficking, FUS can also modulate the TME by inducing sterile inflammation, activating microglia, and recruiting immune cells, thus augmenting CAR-T cell activity.

Advancements in CAR T cell engineering are also pivotal. Gene-editing technologies like CRISPR-Cas9 enable precise modifications to enhance CAR-T cell functionality, including knocking out inhibitory receptors such as PD-1, optimizing metabolic pathways for improved cell fitness, and designing CARs that secrete pro-inflammatory cytokines to reshape the TME. Furthermore, combining CAR-T cells with immune checkpoint inhibitors has shown synergistic potential in reversing T cell exhaustion and boosting therapeutic efficacy.

Meanwhile, ongoing manufacturing innovations are grappling with key bottlenecks in scalability, cost, and quality control. For instance, circRNAs have been explored as potential candidates to alleviate concerns of safety and precision in the context of CAR-T cell therapy (271). Moreover, non-viral gene delivery methods, such as electroporation and nanoparticle-based transfection, are reducing reliance on viral vectors, thereby lowering production costs and enabling precise engineering. Moreover, dynamic bioreactors, feeder-free culture systems, and automation-driven workflows are optimizing CAR-T cell expansion and functionality while expediting production timelines to improve patient accessibility.

Looking forward, these advancements pave the way for more effective and personalized treatment strategies. Real-time imaging technologies, including immunoPET and MRI, will enable dynamic monitoring of CAR-T cell behavior, allowing clinicians to track trafficking, proliferation, and efficacy within the CNS and adjust therapies in real time. Additionally, synergistic approaches, such as combining CAR-T cells with oncolytic viruses or integrating innovative delivery systems like FUS and nanotechnology, are promising strategies to overcome TME-induced resistance and improving therapeutic outcomes. Additionally, the potential incorporation of CAR technology into other cell lines such as macrophages and Natural Killer (NK) will help further define and characterize the obstacles ahead of recombinant adoptive cellular therapies (272).

In conclusion, CAR-T cell therapy for brain tumors has made significant progress, yet critical hurdles remain. Addressing challenges such as trafficking, TME suppression, and persistence through multidisciplinary innovation and engineering is vital for expanding CAR-T cell efficacy in CNS malignancies. With ongoing advancements in gene editing, manufacturing, and combination therapies, CAR-T cell therapy is poised to transform the treatment landscape, offering renewed hope for patients and families affected by these devastating diseases.
